# Plant diversity increases spatio‐temporal niche complementarity in plant‐pollinator interactions

**DOI:** 10.1002/ece3.2026

**Published:** 2016-03-04

**Authors:** Christine Venjakob, Alexandra‐Maria Klein, Anne Ebeling, Teja Tscharntke, Christoph Scherber

**Affiliations:** ^1^ AgroecologyDNPWGeorg‐August‐University GöttingenGöttingenGermany; ^2^ Institute of Ecology, Ecosystem FunctionsLeuphana University of LüneburgLüneburgGermany; ^3^ Faculty of Environment and Natural ResourcesNature Conservation and Landscape EcologyUniversity of FreiburgFreiburgGermany; ^4^ Institute of EcologyFriedrich‐Schiller‐University of JenaJenaGermany; ^5^ Institute of Landscape EcologyUniversity of MünsterMünsterGermany

**Keywords:** Environmental niche, floral resource use, functional pollinator diversity, generalized additive models, Jena Experiment, niche overlap

## Abstract

Ongoing biodiversity decline impairs ecosystem processes, including pollination. Flower visitation, an important indicator of pollination services, is influenced by plant species richness. However, the spatio‐temporal responses of different pollinator groups to plant species richness have not yet been analyzed experimentally. Here, we used an experimental plant species richness gradient to analyze plant–pollinator interactions with an unprecedented spatio‐temporal resolution. We observed four pollinator functional groups (honeybees, bumblebees, solitary bees, and hoverflies) in experimental plots at three different vegetation strata between sunrise and sunset. Visits were modified by plant species richness interacting with time and space. Furthermore, the complementarity of pollinator functional groups in space and time was stronger in species‐rich mixtures. We conclude that high plant diversity should ensure stable pollination services, mediated via spatio‐temporal niche complementarity in flower visitation.

## Introduction

Declining biodiversity has been shown to affect many ecosystem processes, including pollination services (Biesmeijer et al. 2006; Garibaldi et al. 2013). Grassland biodiversity experiments have demonstrated that plant diversity increases pollinator abundance and species richness (Ebeling et al. 2008; Scherber et al. 2010). However, the mechanisms structuring pollinator community structure have remained unclear, and spatio‐temporal pollinator behavior (including foraging behavior; e.g., Rusterholz and Baur 2010) in response to plant diversity has only rarely been addressed.

Plant species richness has been shown to reduce niche overlap among coexisting plant species (von Felten et al. 2009), affecting floral traits (Binkenstein et al. 2013), thereby indirectly structuring pollinator communities (Potts et al. 2003; Ebeling et al. 2008). However, niche overlap has so far rarely been studied from a pollinator's perspective. One reason may be that the classical Hutchinsonian niche concept (Hutchinson 1957; modified by Holt 2009) involves demographic parameters that are usually not measurable for whole pollinator communities. An alternative approach to study niche overlap in pollinator communities is the concept of environmental niches (Tracy and Christian 1986; Chesson et al. 2001), which includes spatio‐temporal niches, where the focus is on an organism's foraging range. Small organisms, such as pollinators, can differ in spatio‐temporal niches that are potentially modified by plant diversity, as predicted by the biodiversity‐niche hypothesis (MacArthur 1955; Loreau et al. 2001; Rosenfeld 2002; Blüthgen and Klein 2011).

Plant diversity may modify the spatio‐temporal niches of pollinators in two ways: first, the number of plant species itself may affect pollinators’ spatio‐temporal resource use; second, plant diversity may affect vegetation parameters (e.g., floral abundance, vegetation structure) or abiotic conditions (e.g., microclimate) that then indirectly affect resource use.

Individual functional groups of pollinators may express phenological and/or architectural niche complementarity (Blüthgen and Klein 2011). Hoehn et al. (2008) observed spatial complementarity of pollinators within small plots of flowering herbaceous pumpkin plants; similar results were reported by Brittain et al. (2013) for woody almond trees. However, studies are lacking where resource diversity (here: plant diversity) has been experimentally manipulated to study pollinators’ environmental niches in space and time at sufficient spatio‐temporal resolution.

Previous studies have used experimental manipulations of plant species richness (Roscher et al. 2004) to investigate plant–pollinator interaction networks in response to plant species richness on a coarse time scale (months or years; Ebeling et al. 2008). These studies demonstrate temporal stability of flower visitation, indicating higher complementarity in time, due to increasing flower cover provided by high richness of flowering plant species.

However, the effects of declining plant species richness on spatio‐temporal dynamics of plant–pollinator interactions have so far never been investigated. While studies investigating plant–pollinator interactions along plant diversity gradients exist (Ebeling et al. 2008; Scherber et al. 2010; Hudewenz et al. 2012), the spatial and/or temporal resolution of these datasets has not allowed deeper insights into spatial complementarity (vegetation strata) or temporal complementarity (time of day) in floral resource use.

Here, we use a large‐scale, long‐term biodiversity experiment (Roscher et al. 2004) to study the effects of plant species richness on spatio‐temporal complementarity of pollinators. Our study provides new insights into the timing of flower visitation, its spatial stratification, and how spatio‐temporal niches are modified by declining plant species richness. The dataset we analyze has an unprecedented spatio‐temporal resolution: almost 12 h of observation in three vegetation strata on *N* = 19 plots across a whole flowering season.

The overall aim of our study was to understand the complexity of niche differentiation in three dimensions (space, time, and plant species richness), across four pollinator functional groups (bumblebees, honeybees, solitary bees, and hoverflies). In particular, we test the following hypotheses:

1. Plant species richness modifies pollinator flower visitation height and timing within a day either directly or indirectly (i.e., modified by vegetation characteristics such as flower cover).
2. Individual pollinator functional groups visit different flowering heights in the vegetation and at different times of day, depending on plant species richness.
3. Species‐rich plant communities are characterized by higher complementarity in spatio‐temporal resource use because of higher resource diversity and higher specialization of pollinators (Blüthgen and Klein 2011).

## Materials and Methods

### Experimental design

**Figure 1:**
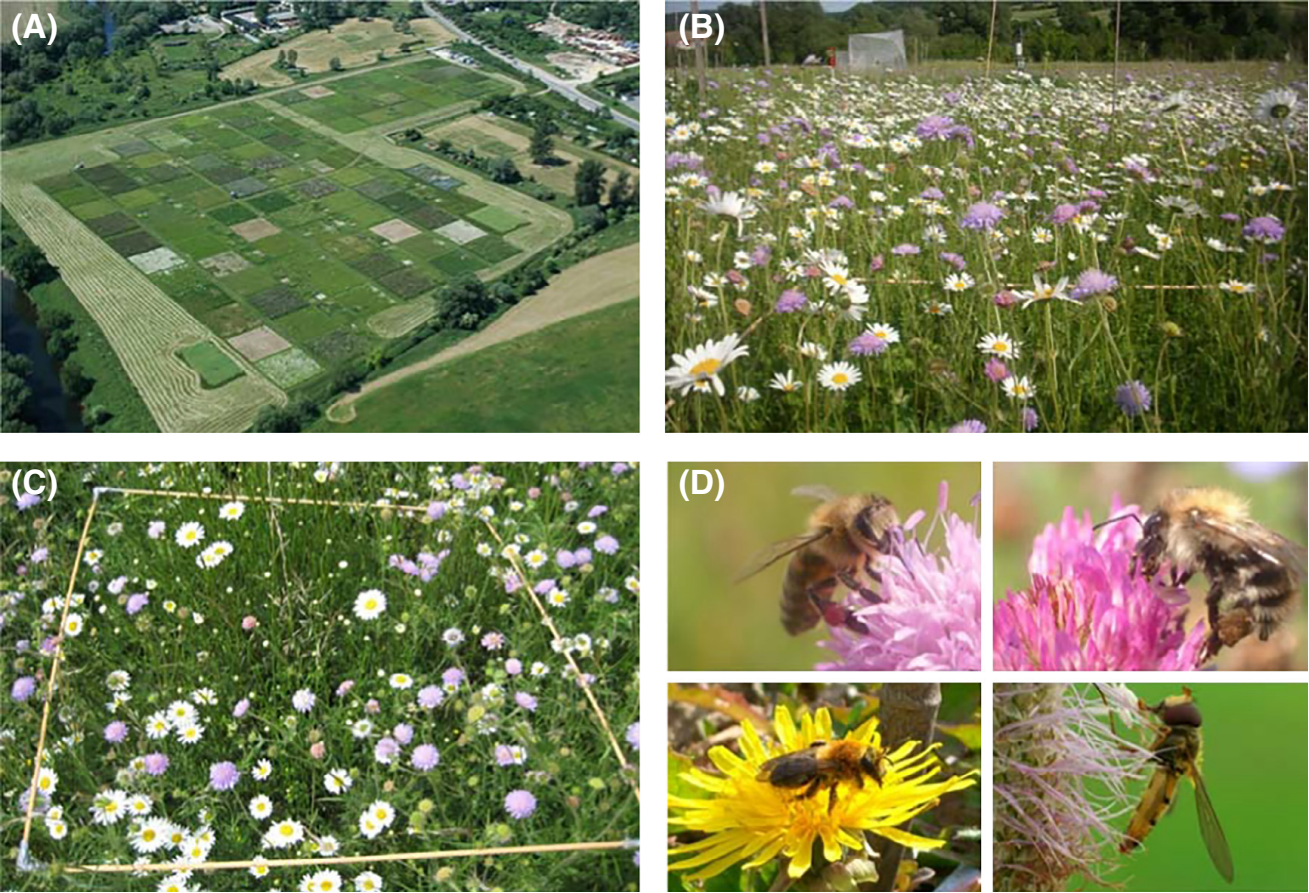
(A) Overview of the Jena Experiment (photograph by The Jena Experiment/C. Scherber/A. Weigelt/W. Voigt), (B) profile view from an example study plot with *Leucanthemum vulgare* and *Knautia arvensis* (photograph by C. Venjakob), (C) Square frame (0.8 m × 0.8 m) representing the sampling area where flower visitors were observed (photograph by C. Venjakob), (D) examples of four different pollinator functional groups, clockwise: Honeybee (*Apis mellifera*), bumblebee (*Bombus pascuorum*), solitary bee (*Anthophora* cf*. plumipes*), hoverfly (*Episyrphus* cf. *balteatus*) (photographs by C. Venjakob).

The study was conducted as part of the Jena Experiment that was established in 2002 to investigate how plant biodiversity affects ecosystem functioning. The field site (Fig. 1A) comprises 10 ha and is located in the north of Jena (Jena‐Löbstedt, Thuringia, Germany), on the flood plain of the river Saale (50°55′N, 11°35′E; 130 m a.s.l.). There are 82 plots divided into two adjacent subplots comprising 6 × 5.5 m and 3 × 3.5 m, respectively (43.5 m^2^ in total). Plant species richness was manipulated by establishing a gradient from 1 to 60 plant species per plot, containing either one, two, four, eight, 16, or 60 plant species from a total pool of 60 plant species (Roscher et al. 2004). Plots with a plant species richness of one, two, four, or eight plant species were replicated 16 times, whereas plots with a plant species richness level of 16 and 60 plant species were replicated 14 and four times, respectively. The 60 plant species were divided into four functional groups (small herbs, tall herbs, legumes, and grasses) using morphological, phenological, and physiological traits (for further details see Roscher et al. 2004). Species within plots were originally sown in equal proportions. Plots were arranged in four blocks, accounting for changes in soil abiotic conditions perpendicular to the river Saale (Roscher et al. 2004). All plots were mown every June and September according to common extensive grassland management. Plots were weeded twice per year until 2009 and afterward three times per year (April, July, and October) to maintain the sown target plant species mixtures.

From the experimental pool of 60 plant species, including 16 grass species that are not insect‐pollinated, 32 entomophilous plant species were previously observed to be visited by functionally relevant insect pollinators (Ebeling et al. 2008). A crucial trait of plant species visited by insects is the development of flowers that produce pollen and nectar. We divided all flowering plant species into three functional groups: small herbs, tall herbs, or legumes (see Table S1).

Because we aimed at detailed, long‐time pollinator observations (resulting in almost 12 h per plot in total), we restricted our observations to a subset of plots defined as follows: (i) no grasses present (except 60‐species mixtures and plots B3A22, B4A11); (ii) >2 plant species present, and (iii) 4 or 5 replicates in each block. This resulted in *N* = 19 plots used in this study (see Fig. S1).

### Pollinator observations

Pollinator observations were conducted from 11 April until 23 August in 2011, with approximately 3 weeks between observations, depending on flower cover and weather conditions. Observation time was from early morning (7–9 am) until evening (6.30–8.30 pm). Sunny conditions with a minimum temperature of 18°C with windless to slightly windy conditions (<2 m/sec) were chosen for the observations.

All 19 plots were observed together on every sampling day according to a synchronized scheme by one observer per block and plot with an additional observer on the observation days 12 July and 2 August.

The observations were performed at the same location for each observation day. A square frame of 0.8 m × 0.8 m was systematically placed inside the central core area of each plot to observe flower‐visiting pollinators (see Fig. 1B,C), on either the eastern or western side of the plot, facing away from main pathways. Distance between plot centroids ranged from 28 to 405 m. Each plot was observed for 15 min, representing a “single observation period”. A fully observed block of five single‐observation periods plus walking time between the plots plus a buffer time of 15 min (5 plots 15 min + 6 · 3 min walking + 15 min buffer = 1 h 48 min) was considered a “run”. After each run, each observer switched to the next block (clockwise). Observation order of plots within a block changed (clockwise) for each new run. Each of the 19 plots was observed up to seven times per day. In total, we observed each plot 47 times on 7 days (total observation time: 11.75 h per plot).

During the observations, all pollinator visits on flowers to the plot were recorded as well as the identity of the visited plant species. Additional to our focal groups (bumblebees, honeybees, solitary bees, and hoverflies), we observed other groups of pollinators such as beetles, wasps (Hymenoptera: Vespidae), flies (Diptera), and ants (Hymenoptera: Formicidae). While we recorded all visitations by these groups, we decided to exclude them from analyses as their abundances were very low and models (e.g., for wasps) did not converge due to lack of sufficient observations. Flower visitation rate was defined as the number of flower visits per plot during a single observation period. Pollinators were identified in the field to genus, morphospecies, or species level, or caught with a sweep net for subsequent identification in the laboratory. Honeybees, bumblebees, solitary bees, and hoverflies (see Fig. 1D) were grouped for further analyses. Solitary bees were defined as non‐Apis bees sensu Brittain et al. (2013).

For each pollinator observation, we documented time of day. Insects clearly not feeding on pollen or nectar were not considered.

Flower visitation height (flowering height) was recorded using three categories: ground level (1–10 cm), intermediate (11–25 cm), and upper vegetation (≥26 cm). Strata were defined following Lorentzen et al. (2008) with a focus on lower vegetation layers (flowering height of small herbs, *reptantia* [sensu Ellenberg and Mueller‐Dombois 1967]: *c*. 10 cm and *rosulata*:* c*. 25 cm).

Percentage flower cover of each plant species was recorded within the frame by estimating percentage of open flowers in relation to the total observed area. These measurements were used to calculate flower cover and realized species richness of flowering plants (see Figs. S2, S3).

### Statistical analyses

#### Calculation of standardized daytime

We calculated a standardized daytime (SDT) ranging from zero to one, based on sunrise and sunset, separately for each day, because day length changed over the year. The full code for this is in the Supporting Information (Appendix S1). SDT was aggregated to 1 significant digit, resulting in nine time steps.

#### Generalized additive mixed models for flower visitation rates

The number of visits for each pollinator group was summed for each plot, time of day, and flowering height, resulting in a sample size of 309. Time of day was aggregated on the basis of hours and minutes. We analyzed the effect of (i) plant species richness (which was the main explanatory variable) and (ii) flower cover (to test for potential effects of resource abundance) on flower visitation rate (number of flower visits; count data) of pollinators using generalized additive mixed models (GAMMs) with negative binomial errors (R, package: mgcv, version 1.7‐28 [Wood 2006]). Additive models allowed us to model three‐way nonlinear interactions, while accounting for spatio‐temporal nonindependence in the data both with smooth terms and random effects. Initial models included either plant species richness or flower cover, and time of day and flowering height as fixed effects, fitted sequentially using smooth terms defined by tensor product interactions as implemented in the ti() function in GAMM. Random effects for plot and height stratum were used. We added stratum as a random effect to account for spatio‐temporal nonindependence of observations taken within the height strata of a particular plot. This is similar to a split‐plot design, where the height strata are the “subplots” within a plot. Fixed‐effects terms were modeled using a basis dimension of *k* = 3 with option “select = T”. The theta parameter of the negative binomial distribution was estimated during model fitting by specifying a starting interval within [0;10]. For further model simplification, we performed backward selection, which was performed by manually removing each term with the highest *P*‐value sequentially (as indicated in summary.gam). We continued refitting the model until all terms were either significant or were part of a higher‐order significant interaction term according to the principle of marginality (see R documentation on gam.selection [Wood 2014]). To compare among different sets of explanatory variables (e.g., flower cover vs. plant species richness), we used Akaike's information criterion with a correction for finite sample sizes (AICc; Scherber et al. 2014; Wood 2014). Explained deviance for each model was calculated as described in the Supporting Information, Appendix S2.

#### Multinomial models for pollinator community composition

Changes in pollinator community composition and spatio‐temporal niche complementarity were assessed using multinomial models with plant species richness, flowering height, and time of day as explanatory variables. This was carried out using a data frame containing the flower visitation rates of each pollinator group as a response matrix (La Rosa et al. 2012; Qian et al. 2012). Explanatory variables were the same as for the GAMMs, but these were fitted as polynomial splines using the bs() function in the splines package in R. Model fitting was performed with the function “multinom” (R, package: nnet, version 7.3‐7 [Venables and Ripley 2002]). Models were simplified using stepAIC (MASS library), and significance of terms was assessed using likelihood ratio tests. Differences in pollinator community composition were assessed by dividing the model coefficients by their standard errors; we then used two‐tailed Wald *z*‐tests to test significance of these coefficients. We additionally refitted multinomial models with random effects using function “BayesX” in R package R2BayesX, version 1.0.0 (Belitz et al. 2015; Umlauf et al. 2015). However, in these models, only linear three‐way interactions were fitted as three‐dimensional smooth terms are not yet implemented.

#### Analysis of niche complementarity versus overlap

To assess whether pollinator groups differed in spatio‐temporal niche complementarity versus overlap, we used the predictions of the GAMM models (see II.) and transformed these into presence/absence data for each group (setting singletons to zero). This resulted in four vectors containing the presences/absences for each pollinator group i (coded as 0 or 1). Using bumblebees as our reference category, we calculated pairwise sums of these vectors to arrive at three resource use categories: 0 (neither bumblebee nor group i present); 1 (either bumblebee or group i present; indicating complementarity); 2 (both bumblebee and group i present; indicating overlap). The resulting vector (consisting of 0's, 1's, and 2's) was then entered into a multinomial model as described above (see III.) to explicitly test for resource use. In these models, plant diversity was entered as a factor, and other terms were entered using natural splines. All analyses were performed using R, version 3.0.1 (R Development Core Team 2013).

## Results

### Flower visitor community

We recorded 59 flower‐visiting species with a total of 10,653 individual flower visits on 34 different flowering plant species. Comprising 31 solitary bee species (including semisocial species) with 236 visits, ten bumblebee species with 3,059 visits, 17 hoverfly species with 676 visits, and the European honey bee (*Apis mellifera* L.) with 6,682 flower visits (see Table S2). Common sainfoin (*Onobrychis viciifolia* Scop.) was the most frequently visited plant species, although it was not more abundant than other flowering species (see Table S3 and Fig. S2); it was mainly visited by honeybees and the red‐tailed bumblebee (*Bombus lapidarius* L.; see Table S3). Other frequently visited plants, mainly visited by honeybees, were field scabious (*Knautia arvensis* (L.) Coult.) and bastard medic (*Medicago* × *varia* Martyn). Meadow crane (*Geranium pratense* L.) and bird's‐foot trefoil (*Lotus corniculatus* L.) were mainly visited by bumblebees, especially by the red‐tailed bumblebee (see Table S3). The most common solitary bee species were *Halictus tumulorum* L., *Lasioglossum calceatum* Scop., *Lasioglossum pauxillum* A. Schenck, which visited mostly meadow crane. Other species frequently visited by solitary bees were germander speedwell (*Veronica chamaedrys* L.) and field scabious; these were visited often either by *Andrena viridescens* Viereck or by *Lasioglossum leucozonium* Schrank, see Table S3.

Hoverflies visited different plant species to bumblebees, honeybees, and solitary bees. They especially favoured hogweed (*Heracleum sphondylium* L.) that was predominantly visited by *Sphaerophoria scripta* L. A sister species of this order (*Sphaerophoria interrupta* Jones) most often visited ribgrass (*Plantago lanceolata* L.). Parsnip (*Pastinaca sativa* L.) was mostly visited by *Melanostoma mellinum* L. (see Table S3).

### Effects of plant species richness, time of day, and flowering height on flower visitation

**Figure 2:**
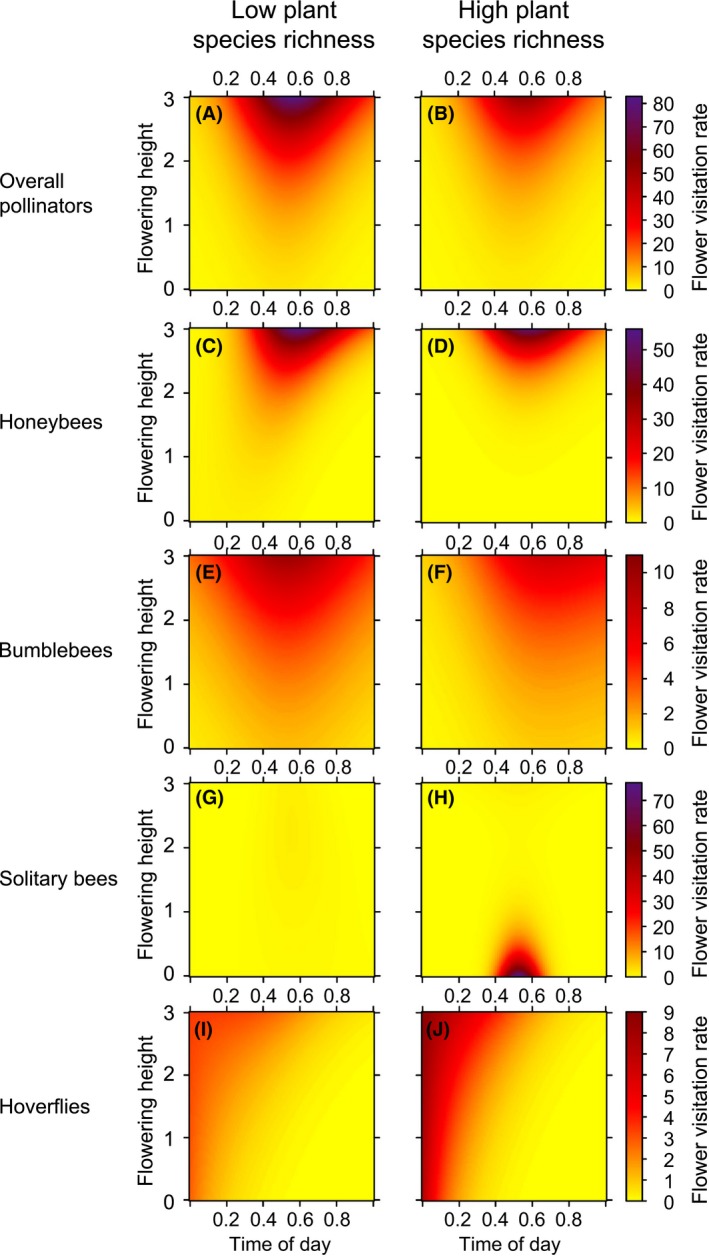
Effects of plant species richness, time of day, and flowering height on flower visitation rate. Shown are the results of minimal adequate generalized additive mixed models, for all pollinator functional groups. (A, B) Overall pollinators (honeybees + bumblebees + solitary bees + hoverflies), and each pollinator group separately (C, D): honeybees, (E, F): bumblebees, (G, H): solitary bees, (I, J): hoverflies. Flower visitation rate is influenced by plant species richness (low = 4, high = 60 plant species), time of day (0–1; range representing the observation time, between the onset of sunrise and sunset), and three different flowering heights (A–J: 1 = 1–10 cm, 2 = 11–25 cm, 3 = ≥26 cm).

Overall flower visitation (i.e., across all pollinator functional groups) showed separate nonlinear (approximately quadratic) effects with time of day and flowering height, resulting in peaks in visitation rates at midday (1–3 pm, i.e., 0.6 standardized time) and in the taller flowers, regardless of plant species richness (Table 1, Fig. 2A,B). When all pollinators, except honeybees, were analyzed, flower visitation rate was not significantly influenced by plant species richness, but there were main effects of flowering height and time of day (Table S4 and Fig. S4).

**Table 1:** Flower visitation rate of the pollinator functional groups: all pollinators, honeybees, bumblebees, solitary bees, and hoverflies. Summary of terms for generalized additive mixed models

Response variable (flower visitation rate)	Parameter	Est. df (est. pp)	Effect^a^	Ref. df (SE)	*F*‐value (*t*‐value)	*P*	Deviance explained [%]
Overall pollinators	(Intercept)	(2.86)	–	(0.11)	(25.51)	<0.001	57
	Time of day^b^	1.88	Quadratic	1.88	47.31	<0.001	
	Flowering height^b^	2.00	Quadratic	2.00	45.49	<0.001	
	Plant species richness^b^	2.00	Quadratic	2.00	1.15	0.319	
	Time of day*Flowering height^b^	1.00	Linear	1.00	2.68	0.103	
Honeybees	(Intercept)	(1.7038)	–	(0.17)	(9.95)	<0.001	66
	Time of day^b^	1.99	Quadratic	1.99	54.67	<0.001	
	Flowering height^b^	2.00	Quadratic	2.00	31.69	<0.001	
	Plant species richness^b^	2.00	Quadratic	2.00	0.89	0.413	
	Time of day*Flowering height^b^	1.00	Linear	1.00	5.82	0.016	
	Time of day*Plant species richness^b^	1.90	Quadratic	1.90	5.00	0.009	
	Flowering height*Plant species richness^b^	1.00	Linear	1.00	1.01	0.316	
	Time of day*Flowering height*Plant species richness^b^	1.87	Quadratic	1.87	4.42	0.015	
Bumblebees	(Intercept)	(1.4597)	–	(0.23)	(6.41)	<0.001	21
	Time of day^b^	1.98	Quadratic	1.98	5.27	0.006	
	Flowering height^b^	2.00	Quadratic	2.00	6.11	0.003	
	Plant species richness^b^	2.00	Quadratic	2.00	0.16	0.851	
	Time of day*Plant species richness^b^	1.00	Linear	1.00	3.94	0.048	
Solitary bees	(Intercept)	(−1.0879)	–	(0.15)	(−7.38)	<0.001	27
	Time of day^b^	1.10	Linear	1.10	50.85	<0.001	
	Flowering height^b^	1.97	Quadratic	1.97	1.70	0.184	
	Plant species richness^b^	2.00	Quadratic	2.00	0.09	0.914	
	Time of day*Flowering height^b^	2.00	Quadratic	2.00	0.05	0.955	
	Time of day*Plant species richness^b^	1.69	Quadratic	1.69	1.55	0.207	
	Flowering height*Plant species richness^b^	2.73	Cubic	2.73	4.66	0.005	
	Time of day*Flowering height*Plant species richness^b^	2.02	Quadratic	2.02	4.37	0.013	
Hoverflies	(Intercept)	(−0.3084)	–	(0.17)	(−1.802)	0.073	41
	Time of day^b^	1.92	Quadratic	1.92	40.85	<0.001	
	Flowering height^b^	2.00	Quadratic	2.00	12.15	<0.001	
	Plant species richness^b^	2.00	Quadratic	2.00	0.08	0.927	
	Time of day*Flowering height^b^	1.00	Linear	1.00	8.73	0.003	
	Time of day*Plant species richness^b^	1.59	Quadratic	1.59	3.84	0.033	

Est.df, estimated degrees of freedom of term; est.pp, estimated parameter value.

a Interpretation of smooth term.

b Term was fitted using ti() function in generalized additive mixed models. *n* = 309.

Honeybee visits were influenced by a three‐way, nonlinear interaction of all three explanatory variables (Table 1, Fig. 2C,D): if plant species richness was low, flower visitation ranged spatially from intermediate flowering height to the upper flowering height and started temporally at about 0.2 standardized time, with a peak at midday (0.5 standardized time, from approx. 11 am–2 pm, depending on the season) and ending in the evening (1 standardized time, from approx. 18–20 pm, depending on the season). By contrast, if plant species richness was high, honeybees limited their spatio‐temporal visitation pattern to the upper flowers, while intermediate flowers were not visited any more. The highest visitation rate was in plant species mixtures with only four plant species, at around midday and in the tallest flowers (Fig. 2D).

Bumblebee visits were influenced by a linear two‐way interaction between plant species richness and time of day (Table 1, Fig. 2E,F): while visitation to species‐poor mixtures showed a peak around midday (0.55 standardized time), visitation shifted toward the evening (0.7 standardized time) in species‐rich mixtures. In addition, there was a separate quadratic effect of flowering height. The interaction and main effects resulted in a height stratification of flower visitation (Fig. 2E,F) with visits increasing toward the top of the vegetation.

Visits of solitary bees were influenced by a nonlinear three‐way interaction among time of day, flowering height, and plant species richness (Table 1, Fig. 2G,H). In species‐poor mixtures, visits were not influenced by time or flowering height, while in species‐rich mixtures visits concentrated near the ground and around midday (depending on the season, approx. 11 am–2 pm; Fig. 2G,H).

Finally, hoverfly visits were influenced by two two‐way interactions between time of day and plant species richness, and by time of day and flowering height (Table 1, Fig. 2I,J). Hoverfly visits occurred preferably early and at high plant species richness; in addition, early visits occurred preferably in the upper vegetation (Fig. 2I,J). Across all plant species richness levels and flowering heights, hoverflies were always the first pollinators that visited the flowers. Independent of plant species richness, hoverflies started visiting flowers equally across all flowering heights in the very early morning. During the course of the day, they concentrated their activity to the intermediate and upper vegetation and later on only to the tallest flowers. Visiting time of the flowers in the bottom and intermediate flowering heights ended about 2 h before midday (approx. 10 am–12 pm, depending on the season).

When flower cover was used as an explanatory variable instead of plant species richness, the model fits were generally poorer for all pollinator groups except solitary bees, as indicated by strong increases in AICc (see Table S5).

### Shifts in pollinator community composition

**Figure 3:**
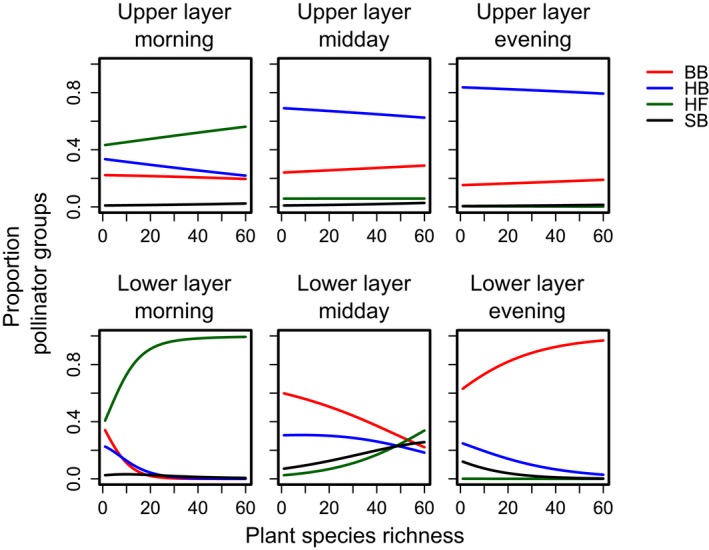
Effects of time of day (morning, midday, and evening), plant species richness (plant species richness: 1–60 plant species), and flowering height (3 = ≥26 cm and 1 = 1–10 cm) on proportions (%) of each flower‐visiting pollinator group [bumblebees [BB = red], honeybees [HB = blue], hoverflies [HF = green], and solitary bees [SB = black]); lines show the predicted values of a minimal adequate multinomial model (likelihood ratio test model with three‐way interactions versus model containing all pairwise two‐way interactions, χ^2^ = 23.058, *P* < 0.001).

To analyze the proportional composition of the pollinator community, we additionally analyzed all groups combined as a multinomially distributed response variable, because responses of pollinator functional groups are likely nonindependent. This resulted in relative data on flower visitation (Fig. 3) analyzed using multinomial models.

These models showed a significant three‐way interaction between plant species richness, flowering height, and time of day (Fig. 3; likelihood ratio = 23.058, *P* < 0.001). There was a strong significant shift in pollinator niches; for example, hoverflies dominated in the morning and at low height, making up almost 100% of the community in species‐rich grassland. However, later in the day, hoverflies became subdominant and were replaced by bumblebees close to ground level. In the tallest vegetation, completely different groups of pollinators dominated the community and they also responded differently to plant species richness. In comparison with the other pollinator functional groups, honeybees were the most frequent flower visitors, making up approximately 60–85% during midday and evening and in the taller vegetation, across all plant species richness levels (Fig. 3). All pollinator groups differed significantly in spatio‐temporal resource use and in their response to plant species richness, as indicated by two‐tailed Wald tests with bumblebees as a reference level (see Table S6).

### Niche complementarity versus overlap

**Figure 4:**
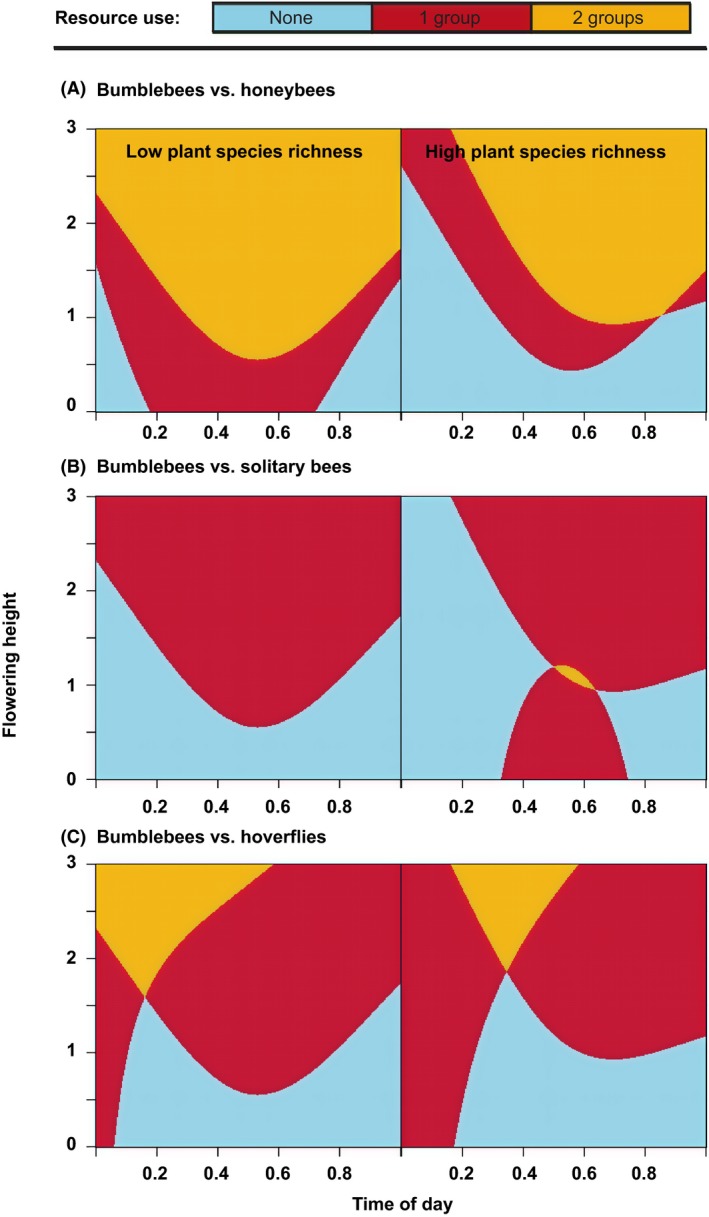
An explicit test of resource use overlap among different functional groups of pollinators. The *x*‐axis represents the standardized time of day (range: 0–1), the *y*‐axis indicates three levels of flowering height (1–3), and the panels show low (4) versus high (60) plant species richness. Colors represent the number of pollinator groups visiting the available plant resources. The Figure shows predictions from multinomial models fitted to categorical response variables, comparing bumblebee presence with the presence of (A) honeybees, (B) solitary bees, and (C) hoverflies.

When analysing resource use (categories 0, 1, or 2), we found significant interactions among plant species richness, flowering height, and time of day for numbers of groups present (Table 2, Fig. 4) for all pollinator groups, indicating significant spatio‐temporal niche shifts. When comparing bumblebees with honeybees (Fig. 4A) and bumblebees with hoverflies (Fig. 4C), we found highly significant three‐way interactions between plant species richness, flowering height, and time of day, indicating that the presence or absence of these groups was modified by space, time, and plant diversity. For solitary bees (Fig. 4B), there were two‐way interactions between time, plant species richness, and flowering height, again indicating strong spatio‐temporal niche shifts.

**Table 2:** Interactions among plant species richness, flowering height, and time of day across all pollinator functional groups (honeybees, bumblebees, solitary bees, and hoverflies). We analyzed pairwise (bumblebees as reference group) presence of all pollinator groups (see text). The response variable was an ordered categorical taking values of 0, 1, or 2 (0 = no pollinator group present, 1 = one pollinator group present, 2 = both pollinator groups present)

Response variable (categorical)	Parameter	LR Chisq	df	Pr (>Chisq)
Bumblebees and honeybees	Flowering height^a^	484.23	2	<0.001
	Time of day^a^	108.32	2	<0.001
	Plant species richness	103.00	2	<0.001
	Flowering height^a^ : Time of day^a^	62.76	2	<0.001
	Flowering height^a^ : Plant species richness	15.86	2	<0.001
	Time of day^a^: Plant species richness	17.40	2	<0.001
	Flowering height^a^ : Time of day^a^: Plant species richness	34.95	2	<0.001
Bumblebees and solitary bees	Flowering height^a^	244.42	2	<0.001
	Time of day^a^	96.31	2	<0.001
	Plant species richness	5.33	2	0.070
	Flowering height^a^: Time of day^a^	62.80	2	<0.001
	Flowering height^a^: Plant species richness	41.87	2	<0.001
	Time of day^a^: Plant species richness	10.29	2	0.006
	Flowering height^a^*Time of day^a^*Plant species richness	1.80	2	0.406
Bumblebees and hoverflies	Flowering height^a^	570.19	2	<0.001
	Time of day^a^	129.63	2	<0.001
	Plant species richness	12.26	2	0.002
	Flowering height^a^: Time of day^a^	38.44	2	<0.001
	Flowering height^a^: Plant species richness	6.48	2	0.039
	Time of day^a^: Plant species richness	29.81	2	<0.001
	Flowering height^a^*Time of day^a^*Plant species richness	25.13	2	<0.001

The results are shown as an analysis of deviance table (Type II tests; LR: likelihood ratio calculated using chi‐square test, df: unused degrees of freedom, Pr(>chi‐square test): *P*‐value).

a Term was fitted in a multinomial model using natural splines.

## Discussion

**Figure 5:**
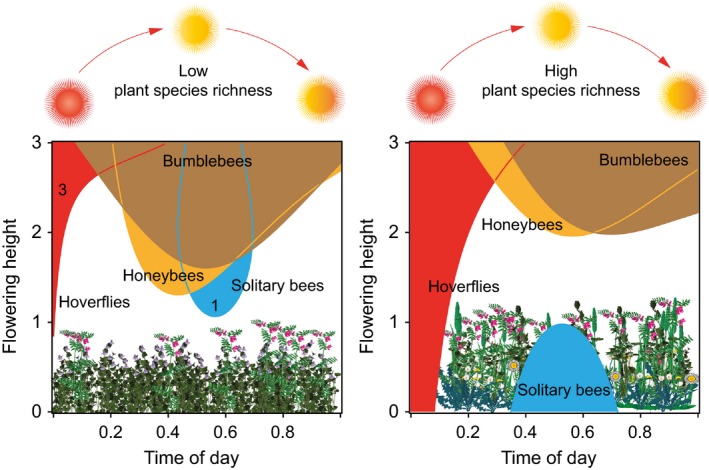
A summary of niche complementarity and overlap in functional groups of pollinators, showing effects of plant species richness, time of day, and flowering height on flower visitation rate of each group. Flower visitation patterns of honeybees (orange), bumblebees (brown), solitary bees (blue), and hoverflies (red) along a plant species richness gradient. The *x*‐axis is standardized time of day (range: 0–1), the *y*‐axis is flowering height (1–3), and the panels show low (4) versus high (60) plant species richness. Pronounced niche complementarity of all pollinator functional groups in species‐rich mixtures (visitation patterns start at four visits, except in low plant species richness mixtures where solitary bees visited only once and hoverflies up to three times [see Fig. 2]). Patterns are predictions from minimal adequate generalized additive mixed models models, created using the ContourPlot function in R (package: lattice, version 0.20‐15 [Sarkar 2008]).

This study demonstrates that experimentally manipulated plant species richness influences spatial and temporal complementarity of flower visitation. We have shown that plant species richness, combined with time and space or with either time or space, drives spatio‐temporal niche complementarity in different and globally relevant pollinator functional groups such as honeybees, bumblebees, solitary bees, and hoverflies (Fig. 5). In our study, overall flower visitation rate was strongly influenced by time or flowering height, but not by plant species richness per se. The pattern remained after excluding honeybees from the model, which corroborates that honeybees were not the main driver of overall flower visitation, despite their high abundance (see Table S4 and Fig. S4). Previous studies (e.g., Ebeling et al. 2008) reported significant main effects of plant species richness on overall flower visitation. Differences between both results could be due to (i) sample sizes/plot selection, (ii) different observation times, or (iii) higher spatio‐temporal resolution. For (i), we reanalyzed data from Ebeling et al. (2008) and restricted these to the *N* = 19 plots used in our study (see Fig. S5). These analyses showed that even with *N* = 19 plots, positive plant species richness effects could have been found. For (ii), we restricted analyses of our data to times of day before 5 pm (as in Ebeling et al. 2008) and again found no differences. For (iii), we excluded all spatio‐temporal information, also resulting in a positive effect of plant species richness. We therefore conclude that differences are due to a higher spatio‐temporal resolution in the current dataset. In addition, competition between pollinators for floral resources is another possible mechanism of resource partitioning (Fründ et al. 2013). While we only indirectly manipulated the availability of floral resources (via plant diversity), our findings indicate that changes in resource availability (flower cover, see Fig. S3) influence spatio‐temporal niche partitioning in different pollinator functional groups (Fontaine et al. 2008; Fründ et al. 2013). Overall flower visitation covered almost the full volume of spatio‐temporal niche space (Fig. 2A,B) (Blüthgen and Klein 2011). In contrast, individual functional groups of pollinators used space and time differently, depending on plant species richness. Other studies have shown that crop monocultures, such as orchards and strawberry or blueberry fields (Carré et al. 2009; Holzschuh et al. 2012; Blaauw and Isaacs 2014; Rosa García and Miñarro 2014), are comparable with species‐poor mixtures that offer limited flower resources in a small volume of niche space. This potentially causes limitations in food supply, for example, because peak flowering time is often limited to a few weeks (Rosa García and Miñarro 2014). Furthermore, the dynamically changing spatio‐temporal flower visitation patterns of some pollinator functional groups indicate stability against biodiversity loss (insurance hypothesis) in species‐rich mixtures. A superposition of pollinator niches (Fig. 5) shows that hoverflies and solitary bees occupied separate spatio‐temporal niches when compared to all other functional groups, while bumblebees and honeybees showed higher niche overlap. Based on the analysis using flower cover as a potential explanatory variable (see Table S5), it is clearly shown that except for solitary bees all pollinator functional groups strongly alter their spatio‐temporal niches in response to plant diversity and in addition to that, plant species richness is a better predictor in combination with time and space than flower cover. Spatio‐temporal niche overlaps are decreasing with increasing plant species richness (Fig. 4). But niche complementarity and partitioning is increasing in the species‐rich mixtures. Even without considering overlapping niches of hoverflies and solitary bees in the species‐poor mixtures (never more than three visits, see Fig. 5), overall niche overlap was always higher in species‐poor mixtures. In species‐rich mixtures, however, resource distribution in space and time is more complex than in species‐poor plant communities, allowing for greater niche diversity (Elmqvist et al. 2003; Finke and Snyder 2008; Cardinale 2011). Our study shows that declining plant species richness is an important factor influencing functional pollinator composition, thereby decreasing complementarity. Changes in the diet breadth of inferior pollinators in response to the loss of dominant pollinators have been reported by Brosi and Briggs (2013).

In our study, pollinator functional groups largely facilitate each other and show increasing complementarity with plant species richness, thus ensuring stable provision of pollination services. However, spatio‐temporal niches in low plant species mixtures were less well covered than in the species‐rich mixtures. Our analyses showed that this was not caused by a lack of available floral resources, because plant species richness was a better predictor for pollinator visitation than flower cover. Hence, our results support the hypothesis that plant species richness indirectly structures spatio‐temporal resource use of pollinators.

Under field conditions, host plants situated in plant species‐poor locations may face extinction in the long term due to the absence of pollinators (Scheper et al. 2014). In this context, future studies should also investigate plant reproductive success accounting for differences in pollination efficiency (Jauker et al. 2012), thereby looking at the effects of pollinator complementarity on plant reproductive success.

In summary, our study shows that declining plant species richness may alter spatio‐temporal resource use of pollinators, leading to higher complementarity of flower visitation in space and time. This has important implications for plant communities in general where a more efficient use of three‐dimensional space and sufficient temporal coverage is important, not only for plant reproduction, but also for crop pollination.

## Conflict of Interest

None declared.

## Supporting information


**Table S1.** Plant species used in the experiment, with an indication of the flowering phenology during the course of a year.
**Figure S1.** Location of study plots.
**Figure S2.** Top 20 highest flower cover per plant species.
**Figure S3.** The number of flowering plant species and flower cover in relation to the number of identified pollinator species and on number of pollinator visits.
**Appendix S1.** R‐code for the calculation of the SDT.
**Appendix S2.** R‐code for the calculation of the proportion of the deviance.
**Table S2.** List of identified pollinators.
**Table S3.** List of visited plant species by all pollinator functional groups, with pollinator species.
**Table S4.** GAMM model of flower visitation rate of all pollinators (bumblebees, solitary bees, hoverflies) excluding honeybees.
**Figure S4.** Effects of plant species richness, time of day and flowering height on flower visitation rate of the pollinator community without honeybees.
**Table S5.** Plant species richness vs. flower cover as explanatory variable.
**Table S6.** All pollinator groups differed significantly in spatio‐temporal resource use and in their response to plant species richness.
**Figure S5.** Effects of plant species richness on the flower visitation of all pollinators (honeybees, bumblebees, solitary bees, hoverflies), (a) based on data from Ebeling et al. (2008) and (b) based on our data.
