# Supplementary material for: Plant diversity increases spatio‐temporal niche complementarity in plant‐pollinator interactions
Source: Ecol Evol. 2016 Mar 4;6(8):2249–61. doi: 10.1002/ece3.2026 (PMC4782262; doi:10.1002/ece3.2026)
Supplement: Supplementary file 1 — Table S1. Plant species used in the experiment, with an indication of the flowering phenology during the course of a year. Figure S1. Location of study plots. Figure S2. Top 20 highest flower cover per plant species. Figure S3. The number of flowering plant species and flower cover in relation to the number of identified pollinator species and on number of pollinator visits. Appendix S1. R‐code for the calculation of the SDT. Appendix S2. R‐code for the calculation of the proportion of the deviance. Table S2. List of identified pollinators. Table S3. List of visited plant species by all pollinator functional groups, with pollinator species. Table S4. GAMM model of flower visitation rate of all pollinators (bumblebees, solitary bees, hoverflies) excluding honeybees. Figure S4. Effects of plant species richness, time of day and flowering height on flower visitation rate of the pollinator community without honeybees. Table S5. Plant species richness vs. flower cover as explanatory variable. Table S6. All pollinator groups differed significantly in spatio‐temporal resource use and in their response to plant species richness. Figure S5. Effects of plant species richness on the flower visitation of all pollinators (honeybees, bumblebees, solitary bees, hoverflies), (a) based on data from Ebeling et al. (2008) and (b) based on our data. [file ECE3-6-2249-s001.docx]

# Supporting Information:

**Plant diversity increases spatio-temporal niche complementarity in plant-pollinator interactions**

Authors: Christine Venjakob^1,2*^, Alexandra-Maria Klein^3^, Anne Ebeling^4^, Teja Tscharntke^1^ and Christoph Scherber^5^

^1^ Agroecology, DNPW, Georg-August-University Göttingen, Göttingen, Germany

^2^ Institute of Ecology, Ecosystem Functions, Leuphana University of Lüneburg, Lüneburg, Germany

^3^ Faculty of Environment and Natural Resources, Nature Conservation and Landscape Ecology, University of Freiburg, Freiburg, Germany

^4^ Institute of Ecology, Friedrich-Schiller-University of Jena, Jena, Germany

^5^ Institute of Landscape Ecology, University of Münster, Münster, Germany

E-mail addresses of all authors: anne.ebeling@uni-jena.de, alexandra.klein@nature.uni-freiburg.de, ttschar@gwdg.de, Christoph.Scherber@uni-muenster.de

* Corresponding author:

E-mail address: Christine.Venjakob@agr.uni-goettingen.de

Table S1: Plant species used in the experiment, with an indication of the flowering phenology during the course of a year

Figure S1: Location of study plots

Figure S2: Top 20 highest flower cover per plant species. Flower cover was measured as proportion of the observed area (0.8 x 0.8 m) for each plant species and plant species level respectively; shown are the calculated flower cover as mean, maximum and minimum.

Figure S3: The number of flowering plant species and flower cover in relation to the number of identified pollinator species and on number of pollinator visits

Appendix S1: R-code for the calculation of the SDT

Appendix S2: R-code for the calculation of the proportion of the deviance

Table S2: List of identified pollinators

Table S3: List of visited plant species by all pollinator functional groups, with pollinator species

Table S4: GAMM model of flower visitation rate of all pollinators (bumblebees, solitary bees, hoverflies) excluding honeybees

Figure S4: Effects of plant species richness, time of day and flowering height on flower visitation rate of the pollinator community without honeybees

Table S5: Plant species richness vs. flower cover as explanatory variable

Table S6: All pollinator groups differed significantly in spatio-temporal resource use and in their response to plant species richness

Figure S5: Effects of plant species richness on the flower visitation of all pollinators (honeybees, bumblebees, solitary bees, hoverflies), (a) based on data from Ebeling *et al.* (2008) and (b) based on our data

## Figure S1:

**Location of study plots**

| 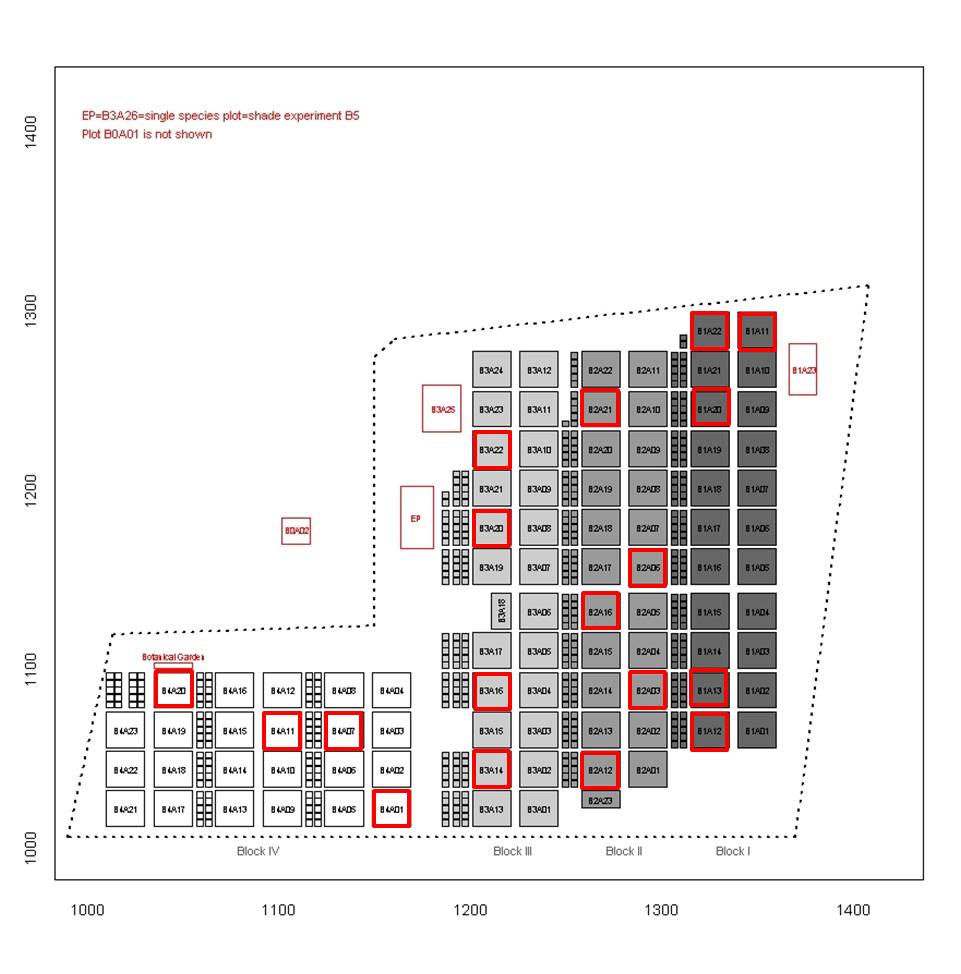  **Figure S1:** Location of individual study plots at the experimental field site. Each block contained up to five plots, except block 3 which contained four plots; red frames mark the plots. |
| --- |

## Table S1: Plant species used in the experiment, with an indication of the flowering phenology over the year.

Table S1: Plant species used in the experiment, with an indication of the flowering phenology over the year. Different functional groups are indicated as leg= legume, therb=tall herbs and sherb=small herbs. Flowering periods for each plant species are marked in orange colour. Pollinator observations were performed for a total of seven days in 2011 (14 April, 5 and 23 May, 23 June, 12 July, 2 and 23 August).

| \| **Plant  species** \| **Family** \| **Funct. Groups (leg, therb, sherb)** \| **January** \| **Febru-ary** \| **March** \| **April** \| **May** \| **June** \| **July** \| **August** \| **Sep-tember** \| **October** \| **Novem-**  **ber** \| **Decem-ber** \| \| --- \| --- \| --- \| --- \| --- \| --- \| --- \| --- \| --- \| --- \| --- \| --- \| --- \| --- \| --- \| \| *Achillea millefolium* \| Asteraceae \| therb \|  \|  \|  \|  \|  \|  \|  \|  \|  \|  \|  \|  \| \| *Ajuga  reptans* \| Lamiaceae \| sherb \|  \|  \|  \|  \|  \|  \|  \|  \|  \|  \|  \|  \| \| *Anthriscus  sylvestris* \| Apiaceae \| therb \|  \|  \|  \|  \|  \|  \|  \|  \|  \|  \|  \|  \| \| *Bellis  perennis* \| Asteraceae \| sherb \|  \|  \|  \|  \|  \|  \|  \|  \|  \|  \|  \|  \| \| *Campanula  patula* \| Campanulaceae \| therb \|  \|  \|  \|  \|  \|  \|  \|  \|  \|  \|  \|  \| \| *Cardamine  pratensis* \| Brassicaceae \| therb \|  \|  \|  \|  \|  \|  \|  \|  \|  \|  \|  \|  \| \| *Carum  carvi* \| Apiaceae \| therb \|  \|  \|  \|  \|  \|  \|  \|  \|  \|  \|  \|  \| \| *Centaurea  jacea* \| Asteraceae \| therb \|  \|  \|  \|  \|  \|  \|  \|  \|  \|  \|  \|  \| \| *Cirsium  oleraceum* \| Asteraceae \| therb \|  \|  \|  \|  \|  \|  \|  \|  \|  \|  \|  \|  \| \| *Crepis  biennis* \| Asteraceae \| therb \|  \|  \|  \|  \|  \|  \|  \|  \|  \|  \|  \|  \| \| *Daucus  carota* \| Apiaceae \| therb \|  \|  \|  \|  \|  \|  \|  \|  \|  \|  \|  \|  \| |
| --- | --- | --- | --- | --- | --- | --- | --- | --- | --- | --- | --- | --- | --- | --- | --- | --- | --- | --- | --- | --- | --- | --- | --- | --- | --- | --- | --- | --- | --- | --- | --- | --- | --- | --- | --- | --- | --- | --- | --- | --- | --- | --- | --- | --- | --- | --- | --- | --- | --- | --- | --- | --- | --- | --- | --- | --- | --- | --- | --- | --- | --- | --- | --- | --- | --- | --- | --- | --- | --- | --- | --- | --- | --- | --- | --- | --- | --- | --- | --- | --- | --- | --- | --- | --- | --- | --- | --- | --- | --- | --- | --- | --- | --- | --- | --- | --- | --- | --- | --- | --- | --- | --- | --- | --- | --- | --- | --- | --- | --- | --- | --- | --- | --- | --- | --- | --- | --- | --- | --- | --- | --- | --- | --- | --- | --- | --- | --- | --- | --- | --- | --- | --- | --- | --- | --- | --- | --- | --- | --- | --- | --- | --- | --- | --- | --- | --- | --- | --- | --- | --- | --- | --- | --- | --- | --- | --- | --- | --- | --- | --- | --- | --- | --- | --- | --- | --- | --- | --- | --- | --- | --- | --- | --- | --- | --- | --- | --- | --- | --- | --- |

(continued)

**Table S1.** (continued)

| \| **Plant  species** \| **Family** \| **Funct. Groups (leg, therb, sherb)** \| **January** \| **Febru-ary** \| **March** \| **April** \| **May** \| **June** \| **July** \| **August** \| **Sep-tember** \| **October** \| **Novem-ber** \| **Decem-ber** \| \| --- \| --- \| --- \| --- \| --- \| --- \| --- \| --- \| --- \| --- \| --- \| --- \| --- \| --- \| --- \| \| *Galium  mollugo* \| Rubiaceae \| therb \|  \|  \|  \|  \|  \|  \|  \|  \|  \|  \|  \|  \| \| *Geranium  pratense* \| Geraniaceae \| therb \|  \|  \|  \|  \|  \|  \|  \|  \|  \|  \|  \|  \| \| *Glechoma  hederacea* \| Lamiaceae \| sherb \|  \|  \|  \|  \|  \|  \|  \|  \|  \|  \|  \|  \| \| *Heracleum  sphondylium* \| Apiaceae \| therb \|  \|  \|  \|  \|  \|  \|  \|  \|  \|  \|  \|  \| \| *Knautia  arvensis* \| Dipsacaceae \| therb \|  \|  \|  \|  \|  \|  \|  \|  \|  \|  \|  \|  \| \| *Lathyrus  pratensis* \| Fabaceae \| leg \|  \|  \|  \|  \|  \|  \|  \|  \|  \|  \|  \|  \| \| *Leontodon  autumnalis* \| Asteraceae \| sherb \|  \|  \|  \|  \|  \|  \|  \|  \|  \|  \|  \|  \| \| *Leontodon  hispidus* \| Asteraceae \| sherb \|  \|  \|  \|  \|  \|  \|  \|  \|  \|  \|  \|  \| \| *Leucanthemum  vulgare* \| Asteraceae \| therb \|  \|  \|  \|  \|  \|  \|  \|  \|  \|  \|  \|  \| \| *Lotus  corniculatus* \| Fabaceae \| leg \|  \|  \|  \|  \|  \|  \|  \|  \|  \|  \|  \|  \| \| *Medicago  lupulina* \| Fabaceae \| leg \|  \|  \|  \|  \|  \|  \|  \|  \|  \|  \|  \|  \| |
| --- | --- | --- | --- | --- | --- | --- | --- | --- | --- | --- | --- | --- | --- | --- | --- | --- | --- | --- | --- | --- | --- | --- | --- | --- | --- | --- | --- | --- | --- | --- | --- | --- | --- | --- | --- | --- | --- | --- | --- | --- | --- | --- | --- | --- | --- | --- | --- | --- | --- | --- | --- | --- | --- | --- | --- | --- | --- | --- | --- | --- | --- | --- | --- | --- | --- | --- | --- | --- | --- | --- | --- | --- | --- | --- | --- | --- | --- | --- | --- | --- | --- | --- | --- | --- | --- | --- | --- | --- | --- | --- | --- | --- | --- | --- | --- | --- | --- | --- | --- | --- | --- | --- | --- | --- | --- | --- | --- | --- | --- | --- | --- | --- | --- | --- | --- | --- | --- | --- | --- | --- | --- | --- | --- | --- | --- | --- | --- | --- | --- | --- | --- | --- | --- | --- | --- | --- | --- | --- | --- | --- | --- | --- | --- | --- | --- | --- | --- | --- | --- | --- | --- | --- | --- | --- | --- | --- | --- | --- | --- | --- | --- | --- | --- | --- | --- | --- | --- | --- | --- | --- | --- | --- | --- | --- | --- | --- | --- | --- | --- | --- |

(continued)

**Table S1.** (continued)

| \| **Plant  species** \| **Family** \| **Funct. Groups (leg, therb, sherb)** \| **January** \| **Febru-ary** \| **March** \| **April** \| **May** \| **June** \| **July** \| **August** \| **Sep-tember** \| **October** \| **Novem-ber** \| **Decem-ber** \| \| --- \| --- \| --- \| --- \| --- \| --- \| --- \| --- \| --- \| --- \| --- \| --- \| --- \| --- \| --- \| \| *Medicago  varia* \| Fabaceae \| leg \|  \|  \|  \|  \|  \|  \|  \|  \|  \|  \|  \|  \| \| *Onobrychis  vicifolia* \| Fabaceae \| leg \|  \|  \|  \|  \|  \|  \|  \|  \|  \|  \|  \|  \| \| *Pastinaca  sativa* \| Apiaceae \| therb \|  \|  \|  \|  \|  \|  \|  \|  \|  \|  \|  \|  \| \| *Pimpinella  major* \| Apiaceae \| therb \|  \|  \|  \|  \|  \|  \|  \|  \|  \|  \|  \|  \| \| *Plantago  lanceolata* \| Plantaginaceae \| sherb \|  \|  \|  \|  \|  \|  \|  \|  \|  \|  \|  \|  \| \| *Plantago  media* \| Plantaginaceae \| sherb \|  \|  \|  \|  \|  \|  \|  \|  \|  \|  \|  \|  \| \| *Primula  veris* \| Primulaceae \| sherb \|  \|  \|  \|  \|  \|  \|  \|  \|  \|  \|  \|  \| \| *Prunella  vulgaris* \| Lamiaceae \| sherb \|  \|  \|  \|  \|  \|  \|  \|  \|  \|  \|  \|  \| \| *Ranunculus  acris* \| Ranunculaceae \| therb \|  \|  \|  \|  \|  \|  \|  \|  \|  \|  \|  \|  \| \| *Ranunculus  repens* \| Ranunculaceae \| sherb \|  \|  \|  \|  \|  \|  \|  \|  \|  \|  \|  \|  \| \| *Rumex  acetosa* \| Polygonaceae \| therb \|  \|  \|  \|  \|  \|  \|  \|  \|  \|  \|  \|  \| |
| --- | --- | --- | --- | --- | --- | --- | --- | --- | --- | --- | --- | --- | --- | --- | --- | --- | --- | --- | --- | --- | --- | --- | --- | --- | --- | --- | --- | --- | --- | --- | --- | --- | --- | --- | --- | --- | --- | --- | --- | --- | --- | --- | --- | --- | --- | --- | --- | --- | --- | --- | --- | --- | --- | --- | --- | --- | --- | --- | --- | --- | --- | --- | --- | --- | --- | --- | --- | --- | --- | --- | --- | --- | --- | --- | --- | --- | --- | --- | --- | --- | --- | --- | --- | --- | --- | --- | --- | --- | --- | --- | --- | --- | --- | --- | --- | --- | --- | --- | --- | --- | --- | --- | --- | --- | --- | --- | --- | --- | --- | --- | --- | --- | --- | --- | --- | --- | --- | --- | --- | --- | --- | --- | --- | --- | --- | --- | --- | --- | --- | --- | --- | --- | --- | --- | --- | --- | --- | --- | --- | --- | --- | --- | --- | --- | --- | --- | --- | --- | --- | --- | --- | --- | --- | --- | --- | --- | --- | --- | --- | --- | --- | --- | --- | --- | --- | --- | --- | --- | --- | --- | --- | --- | --- | --- | --- | --- | --- | --- | --- | --- |

(continued)

**Table S1.** (continued)

| \| **Plant  species** \| **Family** \| **Funct. Groups (leg, therb, sherb)** \| **January** \| **Febru-ary** \| **March** \| **April** \| **May** \| **June** \| **July** \| **August** \| **Sep-tember** \| **October** \| **Novem-ber** \| **Decem-ber** \| \| --- \| --- \| --- \| --- \| --- \| --- \| --- \| --- \| --- \| --- \| --- \| --- \| --- \| --- \| --- \| \| *Sanguisorba  officinalis* \| Rosaceae \| therb \|  \|  \|  \|  \|  \|  \|  \|  \|  \|  \|  \|  \| \| *Taraxacum  officinale* \| Asteraceae \| sherb \|  \|  \|  \|  \|  \|  \|  \|  \|  \|  \|  \|  \| \| *Tragopogon  pratensis* \| Asteraceae \| therb \|  \|  \|  \|  \|  \|  \|  \|  \|  \|  \|  \|  \| \| *Trifolium  campestre* \| Fabaceae \| leg \|  \|  \|  \|  \|  \|  \|  \|  \|  \|  \|  \|  \| \| *Trifolium  dubium* \| Fabaceae \| leg \|  \|  \|  \|  \|  \|  \|  \|  \|  \|  \|  \|  \| \| *Trifolium  fragiferum* \| Fabaceae \| leg \|  \|  \|  \|  \|  \|  \|  \|  \|  \|  \|  \|  \| \| *Trifolium  hybridum* \| Fabaceae \| leg \|  \|  \|  \|  \|  \|  \|  \|  \|  \|  \|  \|  \| \| *Trifolium  pratense* \| Fabaceae \| leg \|  \|  \|  \|  \|  \|  \|  \|  \|  \|  \|  \|  \| \| *Trifolium  repens* \| Fabaceae \| leg \|  \|  \|  \|  \|  \|  \|  \|  \|  \|  \|  \|  \| \| *Veronica chamaedrys* \| Scrophulariaceae \| sherb \|  \|  \|  \|  \|  \|  \|  \|  \|  \|  \|  \|  \| \| *Vicia  cracca* \| Fabaceae \| leg \|  \|  \|  \|  \|  \|  \|  \|  \|  \|  \|  \|  \| |
| --- | --- | --- | --- | --- | --- | --- | --- | --- | --- | --- | --- | --- | --- | --- | --- | --- | --- | --- | --- | --- | --- | --- | --- | --- | --- | --- | --- | --- | --- | --- | --- | --- | --- | --- | --- | --- | --- | --- | --- | --- | --- | --- | --- | --- | --- | --- | --- | --- | --- | --- | --- | --- | --- | --- | --- | --- | --- | --- | --- | --- | --- | --- | --- | --- | --- | --- | --- | --- | --- | --- | --- | --- | --- | --- | --- | --- | --- | --- | --- | --- | --- | --- | --- | --- | --- | --- | --- | --- | --- | --- | --- | --- | --- | --- | --- | --- | --- | --- | --- | --- | --- | --- | --- | --- | --- | --- | --- | --- | --- | --- | --- | --- | --- | --- | --- | --- | --- | --- | --- | --- | --- | --- | --- | --- | --- | --- | --- | --- | --- | --- | --- | --- | --- | --- | --- | --- | --- | --- | --- | --- | --- | --- | --- | --- | --- | --- | --- | --- | --- | --- | --- | --- | --- | --- | --- | --- | --- | --- | --- | --- | --- | --- | --- | --- | --- | --- | --- | --- | --- | --- | --- | --- | --- | --- | --- | --- | --- | --- | --- | --- |

| 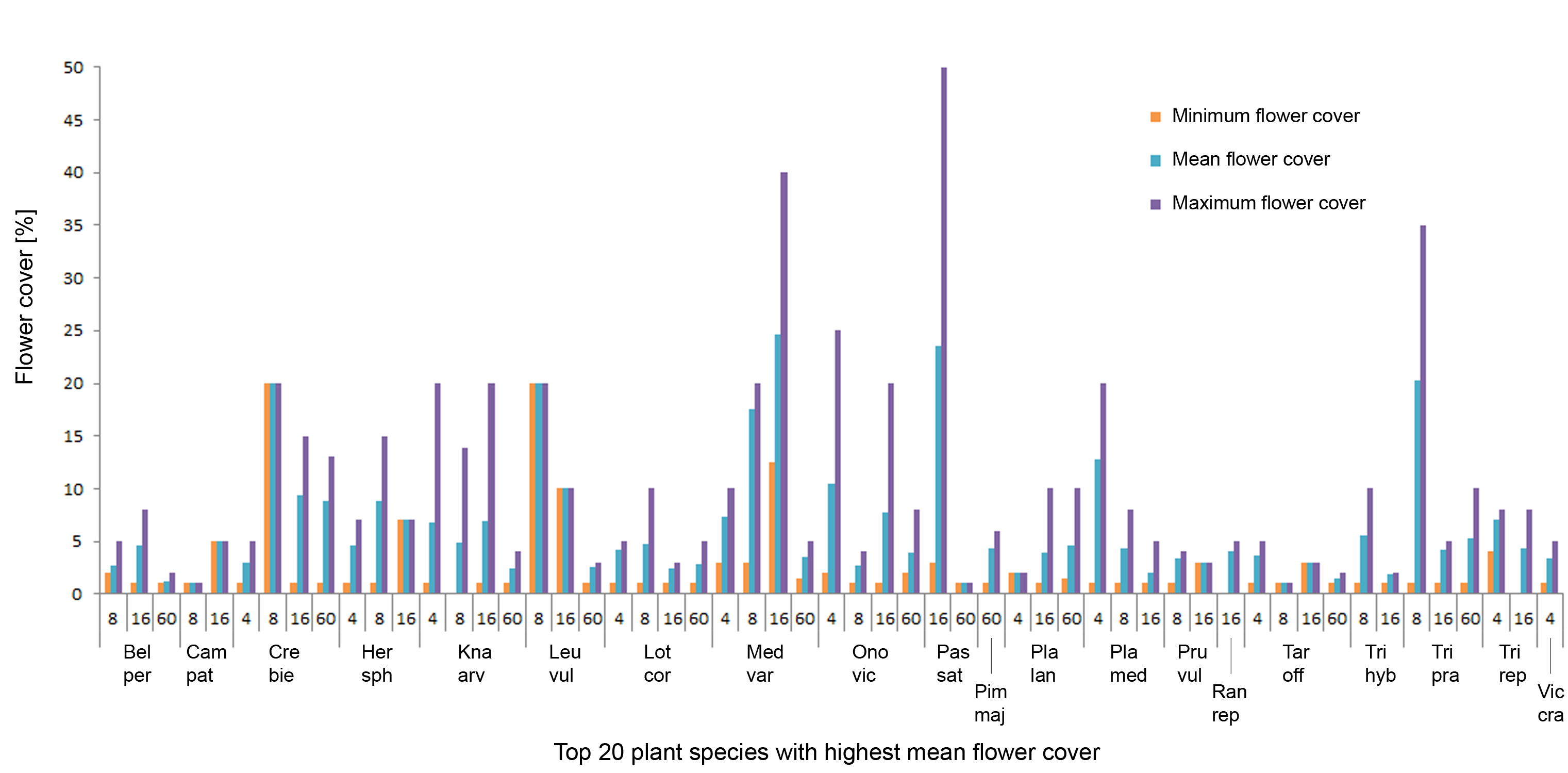  **Figure S2:** Top 20 plant species with the highest mean flower cover (blue) (proportion of observed area) with maximum (lilac) and minimum (orange) flower cover for each plant species (first three letters of the species name were used to form the short version: *Bellis perennis, Campanula patula, Crepis biennis, Heracleum sphondylium, Knautia arvensis, Leucanthemum vulgare, Lotus corniculatus, Medicago varia, Onobrychis vicifolia, Pastinaca sativa, Pimpinella major, Plantago lanceolata, Plantago media, Prunella vulgaris, Ranunculus repens, Taraxacum officinale, Trifolium hybridum, Trifolium pratense, Trifolium repens, Vicia cracca*) and plant species richness level (4, 8, 16, 60) respectively. |
| --- |

## Figure S2: Top 20 plant species with the highest mean flower cover

## Figure S3: Number of pollinator species (a, b) and number of pollinator visits

| (a) (b)  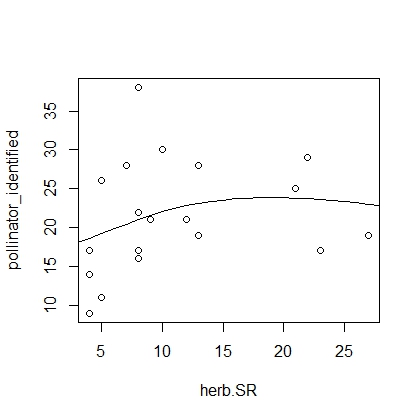 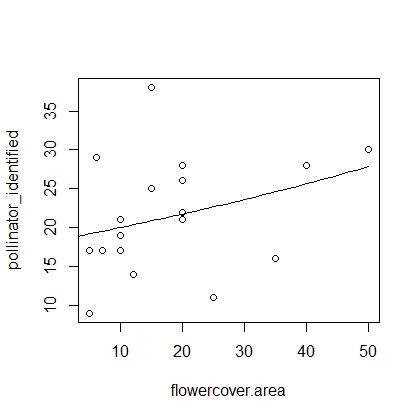  Number of pollinators  Number of flowering plant species Flower cover (%)  (c) (d)  Number of flower visits  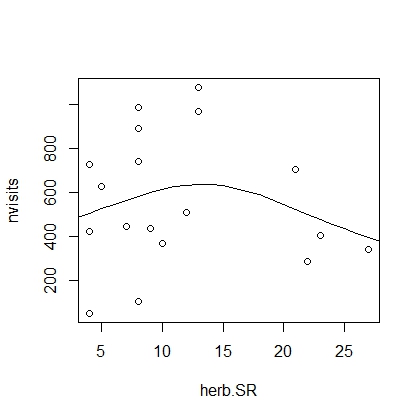 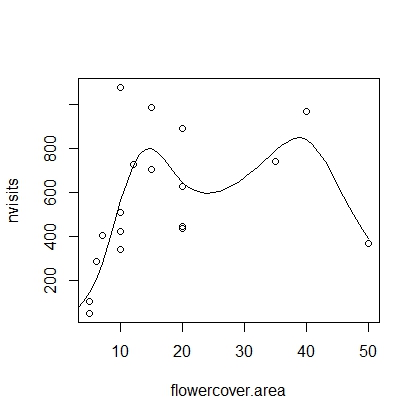  Number of flowering plant species Flower cover (%) |
| --- |
| **Figure S3:** Number of pollinator species (a, b) and number of pollinator visits (c, d) as a function of (realised) number of plant species richness and flower cover. |

## Appendix S1: R-code for the calculation of STD (standardised time)

(R package maptools, version 0.8-34, (Bivand & Lewin-Koh 2015))

library(maptools)

sunrise.set <- function(lat, long, date, timezone="CET", num.days=1){

lat.long <- matrix(c(long, lat), nrow=1)

day <- as.POSIXct(date, tz=timezone)

sequence <- seq(from=day, length.out=num.days , by="days")

sunrise <- sunriset(lat.long, sequence, direction="sunrise",

POSIXct=TRUE)

sunset <- sunriset(lat.long, sequence, direction="sunset", POSIXct=TRUE)

ss <- data.frame(sunrise, sunset)

ss <- ss[,-c(1,3)]

colnames(ss)<-c("sunrise", "sunset")

return(ss)

}

sunrise.set <- function(lat, long, date, timezone="CET", num.days=1){

#this needs to be long lat#

lat.long <- matrix(c(long, lat), nrow=1)

day <- as.POSIXct(date, tz=timezone)

sequence <- seq(from=day, length.out=num.days , by="days")

sunrise <- sunriset(lat.long, sequence, direction="sunrise",

POSIXct=TRUE)

sunset <- sunriset(lat.long, sequence, direction="sunset", POSIXct=TRUE)

ss <- list(sunrise=sunrise, sunset=sunset)

return(ss)

}

mysunset=function(x)

as.POSIXct(

unlist(sunriset(matrix(c(11.624744,50.950989),nrow=1), as.POSIXct(x, tz="CET"), direction="sunset", POSIXct.out=T)[2]),origin="1970-01-01")

mysunrise=function(x)

as.POSIXct(

unlist(sunriset(matrix(c(11.624744,50.950989),nrow=1), as.POSIXct(x, tz="CET"), direction="sunrise", POSIXct.out=T)[2]),origin="1970-01-01")

mysunrises=sapply(B2a$DATE,function(x)mysunrise(x))

mysunsets=sapply(B2a$DATE,function(x)mysunset(x))

sunrise.hour=sapply(mysunrises,function(x)as.POSIXlt(x,origin="1970-01-01")$hour)

sunrise.minute=sapply(mysunrises,function(x)as.POSIXlt(x,origin="1970-01-01")$min)

sunset.hour=sapply(mysunsets,function(x)as.POSIXlt(x,origin="1970-01-01")$hour)

sunset.minute=sapply(mysunsets,function(x)as.POSIXlt(x,origin="1970-01-01")$min)

# convert times to angles using circular distributions

circletime=function(x)(360*x)/24

myradians=function(x)(pi/180)*x

B2a$TIME.decimal=hours(B2a$TIME)+minutes(B2a$TIME)/60

sunrise.dec=sunrise.hour+sunrise.minute/60

sunset.dec=sunset.hour+sunset.minute/60

measured.time=B2a$TIME.decimal

prop.time=function(t1,t2,t3)(t2-t1)/(t3-t1)

## Appendix S2: R-code for the calculation of the proportion of explained deviance (Bolker 2013; Robinson 2013)

The following R code was used (DN=null deviance, DR=residual deviance, DE=explained deviance and DE%= proportion of DE):

DN <- sum(residuals(GAMM-null-model$gam, type="deviance")^2)

DR <- sum(residuals(GAMM-best-model$gam, type="deviance")^2)

DE% <- (DN-DR)*100/DN

## Table S2: Total number of species and flower visits for all functional groups investigated

| **Pollinator groups** | **Total number of**  **species** | **Total number of flower visits** |
| --- | --- | --- |
| **bumblebees** | 10 | 3059 |
| **honeybees** | 1 | 6682 |
| **hoverflies** | 17 | 676 |
| **solitary bees** | 31 | 236 |

## Table S3: List of plant species visited by all pollinator functional groups, with pollinator species.

| **Plant species visited for each pollinator functional group and pollinator species** | **Sum of visits** |
| --- | --- |
| ***Ajuga reptans*** | **20** |
| **bumblebees** | **20** |
| *Bombus lapidarius* | 5 |
| *Bombus pascuorum* | 9 |
| *Bombus terrestris* | 6 |
| ***Bellis perennis*** | **10** |
| **bumblebees** | **2** |
| *Bombus lapidarius* | 2 |
| **hoverflies** | **4** |
| *Sphaerophoria scripta* | 4 |
| **solitary bees** | **4** |
| *Andrena nigroaenea* | 2 |
| *Andrena viridescens* | 1 |
| *Lasioglossum calceatum* | 1 |
| ***Campanula patula*** | **11** |
| **bumblebees** | **4** |
| *Bombus lapidarius* | 4 |
| **hoverflies** | **1** |
| *syrphidae,* unidentified | 1 |
| **solitary bees** | **6** |
| *Lasioglossum calceatum* | 1 |
| *Lasioglossum lativentre* | 3 |
| *Lasioglossum pauxillum* | 1 |
| solitary bee, unidentified | 1 |
| ***Cardamine pratensis*** | **1** |
| **honeybees** | **1** |
| *Apis mellifera* | 1 |
| ***Centaurea jacea*** | **27** |
| **bumblebees** | **2** |
| *Bombus lapidarius* | 1 |
| *Bombus terrestris* | 1 |
| **honeybees** | **23** |
| *Apis mellifera* | 23 |
| **solitary bees** | **2** |
| *Halictus langobardicus* | 1 |
| *Lasioglossum leucozonium* | 1 |
|  | (continued) |
| **Table S3.** (continued) |  |
| **Plant species visited for each pollinator functional group and pollinator species** | **Sum of visits** |
| ***Cirsium oleraceum*** | **1** |
| **bumblebees** | **1** |
| *Bombus quadricolor* | 1 |
| ***Crepis biennis*** | **321** |
| **bumblebees** | **177** |
| *Bombus lapidarius* | 177 |
| **honeybees** | **129** |
| *Apis mellifera* | 129 |
| **hoverflies** | **3** |
| *Episyrphus balteatus* | 1 |
| *Sphaerophoria scripta* | 2 |
| **solitary bees** | **12** |
| *Anthophora aestivalis* | 2 |
| *Lasioglossum calceatum* | 1 |
| *Lasioglossum lativentre* | 2 |
| *Lasioglossum pauxillum* | 1 |
| *Lasioglossum villosulum* | 2 |
| solitary bee, unidentified | 4 |
| ***Galium mollugo*** | **19** |
| **honeybees** | **1** |
| *Apis mellifera* | 1 |
| **hoverflies** | **17** |
| *Episyrphus balteatus* | 8 |
| *Sphaerophoria scripta* | 1 |
| syrphidae*,* unidentified | 8 |
| **solitary bees** | **1** |
| *Halictus tumulorum* | 1 |
| ***Geranium pratense*** | **896** |
| **bumblebees** | **432** |
| *Bombus lapidarius* | 355 |
| *Bombus ruderarius* | 6 |
| *Bombus* sp. | 1 |
| *Bombus terrestris* | 68 |
| *Bombus veteranus* | 2 |
| **honeybees** | **413** |
| *Apis mellifera* | 413 |
| **hoverflies** | **18** |
| *Episyrphus balteatus* | 2 |
|  | (continued) |
| **Table S3.** (continued) |  |
| **Plant species visited for each pollinator functional group and pollinator species** | **Sum of visits** |
| *Eupeodes corollae* | 1 |
| *Melanostoma mellinum* | 4 |
| *Sphaerophoria interrupta* | 1 |
| *Sphaerophoria scripta* | 1 |
| syrphidae, unidentified | 9 |
| **solitary bees** | **33** |
| *Halictus confusus* | 1 |
| *Halictus tumulorum* | 6 |
| *Hylaeus hyalinatus* | 1 |
| *Hylaeus paulus* | 2 |
| *Lasioglossum albipes* | 1 |
| *Lasioglossum calceatum* | 4 |
| *Lasioglossum fulvicorne* | 1 |
| *Lasioglossum laticeps* | 2 |
| *Lasioglossum leucozonium* | 1 |
| *Lasioglossum pauxillum* | 4 |
| *Melitta leporina* | 1 |
| solitary bee, unidentified | 9 |
| ***Glechoma hederacea*** | **11** |
| **bumblebees** | **1** |
| *Bombus lapidarius* | 1 |
| **honeybees** | **1** |
| *Apis mellifera* | 1 |
| **solitary bees** | **9** |
| *Andrena haemorrhoa* | 1 |
| *Andrena mitis* | 1 |
| *Anthophora crinipes* | 2 |
| *Anthophora plumipes* | 3 |
| *Eucera nigrescens* | 2 |
| ***Heracleum sphondylium*** | **213** |
| **honeybees** | **22** |
| *Apis mellifera* | 22 |
| **hoverflies** | **185** |
| *Episyrphus balteatus* | 20 |
| *Eristalis arbustorum* | 1 |
| *Eristalis tenax* | 1 |
| *Eupeodes corollae* | 1 |
| *Melanostoma mellinum* | 36 |
|  | (continued) |
| **Table S3.** (continued) |  |
| **Plant species visited for each pollinator functional group and pollinator species** | **Sum of visits** |
| *Myathropa florea* | 2 |
| *Scaeva pyrastri* | 4 |
| *Sphaerophoria interrupta* | 34 |
| *Sphaerophoria scripta* | 53 |
| *Syritta pipiens* | 2 |
| syrphidae, unidentified | 30 |
| *Syrphus ribesii* | 1 |
| **solitary bees** | **6** |
| *Lasioglossum leucozonium* | 3 |
| solitary bee, unidentified | 3 |
| ***Knautia arvensis*** | **1128** |
| **bumblebees** | **174** |
| *Bombus lapidarius* | 131 |
| *Bombus pascuorum* | 4 |
| *Bombus pratorum* | 9 |
| *Bombus soroeensis* | 2 |
| *Bombus* sp. | 3 |
| *Bombus sylvarum* | 1 |
| *Bombus terrestris* | 23 |
| *Bombus veteranus* | 1 |
| **honeybees** | **878** |
| *Apis mellifera* | 878 |
| **hoverflies** | **52** |
| *Episyrphus balteatus* | 16 |
| *Eristalis jugorum* | 2 |
| *Eristalis tenax* | 4 |
| *Melanostoma mellinum* | 8 |
| *Scaeva pyrastri* | 2 |
| *Sphaerophoria interrupta* | 1 |
| *Syritta pipiens* | 1 |
| syrphidae, unidentified | 18 |
| **solitary bees** | **24** |
| *Andrena cineraria* | 1 |
| *Andrena hattorfiana* | 3 |
| *Halictus scabiosae* | 1 |
| *Lasioglossum calceatum* | 2 |
| *Lasioglossum leucozonium* | 8 |
| *Lasioglossum villosulum* | 1 |
|  | (continued) |
| **Table S3.** (continued) |  |
| **Plant species visited for each pollinator functional group and pollinator species** | **Sum of visits** |
| solitary bee, unidentified | 8 |
| ***Lathyrus pratensis*** | **75** |
| **bumblebees** | **17** |
| *Bombus lapidarius* | 10 |
| *Bombus sylvarum* | 7 |
| **honeybees** | **50** |
| *Apis mellifera* | 50 |
| **solitary bees** | **8** |
| *Halictus confusus* | 2 |
| solitary bee, unidentified | 6 |
| ***Leontodon autumnalis*** | **5** |
| **honeybees** | **3** |
| *Apis mellifera* | 3 |
| **hoverflies** | **2** |
| *Eristalis tenax* | 1 |
| *Sphaerophoria scripta* | 1 |
| ***Leontodon hispidus*** | **7** |
| **bumblebees** | **1** |
| *Bombus lapidarius* | 1 |
| **honeybees** | **2** |
| *Apis mellifera* | 2 |
| **hoverflies** | **4** |
| *Eupeodes corollae* | 1 |
| *Sphaerophoria scripta* | 2 |
| syrphidae, unidentified | 1 |
| ***Leucanthemum vulgare*** | **9** |
| **bumblebees** | **4** |
| *Bombus lapidarius* | 4 |
| **honeybees** | **2** |
| *Apis mellifera* | 2 |
| **hoverflies** | **2** |
| *Episyrphus balteatus* | 2 |
| **solitary bees** | **1** |
| *Andrena flavipes* | 1 |
| ***Lotus corniculatus*** | **538** |
| **bumblebees** | **391** |
| *Bombus lapidarius* | 382 |
| *Bombus pascuorum* | 3 |
|  | (continued) |
| **Table S3.** (continued) |  |
| **Plant species visited for each pollinator functional group and pollinator species** | **Sum of visits** |
| *Bombus* sp. | 1 |
| *Bombus terrestris* | 3 |
| *Bombus veteranus* | 2 |
| **honeybees** | **116** |
| *Apis mellifera* | 116 |
| **hoverflies** | **13** |
| *Episyrphus balteatus* | 3 |
| *Sphaerophoria scripta* | 5 |
| syrphidae, unidentified | 5 |
| **solitary bees** | **18** |
| *Halictus tumulorum* | 8 |
| solitary bee, unidentified | 10 |
| ***Medicago lupulina*** | **5** |
| **hoverflies** | **4** |
| *Sphaerophoria scripta* | 3 |
| syrphidae, unidentified | 1 |
| **solitary bees** | **1** |
| *Lasioglossum fulvicorne* | 1 |
| ***Medicago varia*** | **787** |
| **bumblebees** | **2** |
| *Bombus pascuorum* | 2 |
| **honeybees** | **763** |
| *Apis mellifera* | 763 |
| **hoverflies** | **4** |
| *Episyrphus balteatus* | 1 |
| syrphidae, unidentified | 3 |
| **solitary bees** | **18** |
| *Melitta leporina* | 14 |
| solitary bee, unidentified | 4 |
| ***Onobrychis vicifolia*** | **3947** |
| **bumblebees** | **970** |
| *Bombus lapidarius* | 943 |
| *Bombus pascuorum* | 8 |
| *Bombus ruderarius* | 3 |
| *Bombus* sp. | 8 |
| *Bombus sylvarum* | 1 |
| *Bombus terrestris* | 6 |
| *Bombus veteranus* | 1 |
|  | (continued) |
| **Table S3.** (continued) |  |
| **Plant species visited for each pollinator functional group and pollinator species** | **Sum of visits** |
| **honeybees** | **2934** |
| *Apis mellifera* | 2934 |
| **hoverflies** | **29** |
| *Episyrphus balteatus* | 12 |
| *Eristalis arbustorum* | 1 |
| *Melanostoma mellinum* | 1 |
| *Sphaerophoria scripta* | 4 |
| syrphidae, unidentified | 11 |
| **solitary bees** | **14** |
| *Chelostoma* sp. | 2 |
| *Halictus tumulorum* | 2 |
| *Lasioglossum pauxillum* | 3 |
| *Megachile* sp. | 1 |
| *Melitta leporina* | 2 |
| solitary bee, unidentified | 4 |
| ***Pastinaca sativa*** | **77** |
| **honeybees** | **4** |
| *Apis mellifera* | 4 |
| **hoverflies** | **71** |
| *Chrysotoxum bicinctum* | 1 |
| *Episyrphus balteatus* | 12 |
| *Melanostoma mellinum* | 22 |
| *Melanostoma scalare* | 1 |
| *Scaeva pyrastri* | 2 |
| *Sphaerophoria interrupta* | 1 |
| *Sphaerophoria scripta* | 18 |
| syrphidae, unidentified | 14 |
| **solitary bees** | **2** |
| *Andrena minutuloides* | 1 |
| *Lasioglossum interruptum* | 1 |
| ***Pimpinella major*** | **51** |
| **hoverflies** | **43** |
| *Chrysotoxum bicinctum* | 2 |
| *Episyrphus balteatus* | 9 |
| *Melanostoma mellinum* | 4 |
| *Melanostoma scalare* | 1 |
| *Sphaerophoria interrupta* | 15 |
| *Sphaerophoria scripta* | 6 |
|  | (continued) |
| **Table S3.** (continued) |  |
| **Plant species visited for each pollinator functional group and pollinator species** | **Sum of visits** |
| *Syritta pipiens* | 1 |
| syrphidae, unidentified | 5 |
| **solitary bees** | **8** |
| *Andrena minutula* | 3 |
| *Lasioglossum laticeps* | 2 |
| solitary bee, unidentified | 3 |
| ***Plantago lanceolata*** | **112** |
| **bumblebees** | **1** |
| *Bombus lapidarius* | 1 |
| **honeybees** | **2** |
| *Apis mellifera* | 2 |
| **hoverflies** | **107** |
| *Episyrphus balteatus* | 18 |
| *Melanostoma mellinum* | 16 |
| *Melanostoma scalare* | 1 |
| *Platycheirus parmatus* | 3 |
| *Scaeva pyrastri* | 4 |
| *Sphaerophoria interrupta* | 26 |
| *Sphaerophoria scripta* | 6 |
| syrphidae, unidentified | 32 |
| *Syrphus ribesii* | 1 |
| **solitary bees** | **2** |
| *Lasioglossum calceatum* | 1 |
| solitary bee, unidentified | 1 |
| ***Plantago media*** | **311** |
| **bumblebees** | **43** |
| *Bombus lapidarius* | 7 |
| *Bombus terrestris* | 36 |
| **honeybees** | **202** |
| *Apis mellifera* | 202 |
| **hoverflies** | **60** |
| *Chrysotoxum bicinctum* | 1 |
| *Chrysotoxum cautum* | 2 |
| *Episyrphus balteatus* | 7 |
| *Eristalis tenax* | 1 |
| *Melanostoma mellinum* | 18 |
| *Scaeva pyrastri* | 12 |
| *Sphaerophoria interrupta* | 4 |
|  | (continued) |
| **Table S3.** (continued) |  |
| **Plant species visited for each pollinator functional group and pollinator species** | **Sum of visits** |
| *Sphaerophoria scripta* | 5 |
| syrphidae, unidentified | 10 |
| **solitary bees** | **6** |
| *Andrena hattorfiana* | 1 |
| *Halictus tumulorum* | 1 |
| *Lasioglossum pauxillum* | 4 |
| ***Primula veris*** | **9** |
| **bumblebees** | **4** |
| *Bombus terrestris* | 4 |
| **solitary bees** | **5** |
| *Anthophora plumipes* | 3 |
| *Anthophora spec* | 2 |
| ***Prunella vulgaris*** | **73** |
| **bumblebees** | **66** |
| *Bombus lapidarius* | 64 |
| *Bombus pascuorum* | 1 |
| *Bombus* sp. | 1 |
| **hoverflies** | **7** |
| *Sphaerophoria interrupta* | 1 |
| *Sphaerophoria scripta* | 1 |
| *Syritta pipiens* | 1 |
| syrphidae, unidentified | 4 |
| ***Ranunculus acris*** | **5** |
| **honeybees** | **1** |
| *Apis mellifera* | 1 |
| **hoverflies** | **3** |
| *Episyrphus balteatus* | 1 |
| syrphidae, unidentified | 2 |
| **solitary bees** | **1** |
| *Lasioglossum calceatum* | 1 |
| ***Ranunculus repens*** | **2** |
| **hoverflies** | **1** |
| syrphidae, unidentified | 1 |
| **solitary bees** | **1** |
| *Andrena viridescens* | 1 |
| ***Rumex acetosa*** | **5** |
| **hoverflies** | **5** |
| *Episyrphus balteatus* | 1 |
|  | (continued) |
| **Table S3.** (continued) |  |
| **Plant species visited for each pollinator functional group and pollinator species** | **Sum of visits** |
| *Melanostoma mellinum* | 1 |
| *Sphaerophoria scripta* | 1 |
| syrphidae, unidentified | 2 |
| ***Taraxacum officinale*** | **8** |
| **honeybees** | **8** |
| *Apis mellifera* | 8 |
| ***Trifolium hybridum*** | **256** |
| **bumblebees** | **37** |
| *Bombus lapidarius* | 37 |
| **honeybees** | **210** |
| *Apis mellifera* | 210 |
| **hoverflies** | **8** |
| *Episyrphus balteatus* | 1 |
| *Eristalis interrupta* | 2 |
| syrphidae, unidentified | 5 |
| **solitary bees** | **1** |
| *Halictus eurygnathus* | 1 |
| ***Trifolium pratense*** | **646** |
| **bumblebees** | **312** |
| *Bombus humilis* | 2 |
| *Bombus lapidarius* | 287 |
| *Bombus pascuorum* | 11 |
| *Bombus sylvarum* | 7 |
| *Bombus terrestris* | 5 |
| **honeybees** | **313** |
| *Apis mellifera* | 313 |
| **hoverflies** | **14** |
| *Episyrphus balteatus* | 2 |
| *Melanostoma mellinum* | 5 |
| *Sphaerophoria scripta* | 1 |
| syrphidae, unidentified | 6 |
| **solitary bees** | **7** |
| *Andrena wilkella* | 2 |
| *Halictus eurygnathus* | 2 |
| *Lasioglossum lativentre* | 1 |
| solitary bee, unidentified | 2 |
| ***Trifolium repens*** | **837** |
| **bumblebees** | **376** |
|  | (continued) |
| **Table S3.** (continued) |  |
| **Plant species visited for each pollinator functional group and pollinator species** | **Sum of visits** |
| *Bombus humilis* | 1 |
| *Bombus lapidarius* | 373 |
| *Bombus sylvarum* | 1 |
| *Bombus terrestris* | 1 |
| **honeybees** | **435** |
| *Apis mellifera* | 435 |
| **hoverflies** | **17** |
| *Sphaerophoria interrupta* | 5 |
| *Sphaerophoria scripta* | 8 |
| syrphidae, unidentified | 4 |
| **solitary bees** | **9** |
| *Andrena flavipes* | 3 |
| solitary bee, unidentified | 6 |
| ***Veronica chamaedrys*** | **27** |
| **hoverflies** | **1** |
| syrphidae, unidentified | 1 |
| **solitary bees** | **26** |
| *Andrena viridescens* | 16 |
| *Halictus tumulorum* | 1 |
| solitary bee, unidentified | 9 |
| ***Vicia cracca*** | **203** |
| **bumblebees** | **22** |
| *Bombus humilis* | 2 |
| *Bombus lapidarius* | 4 |
| *Bombus pascuorum* | 15 |
| *Bombus* sp. | 1 |
| **honeybees** | **169** |
| *Apis mellifera* | 169 |
| **hoverflies** | **1** |
| *Episyrphus balteatus* | 1 |
| **solitary bees** | **11** |
| *Lasioglossum fulvicorne* | 10 |
| solitary bee, unidentified | 1 |
| **Total** | **10653** |

## Table S4: GAMM model of flower visitation rate of all pollinators (bumblebees, solitary bees, hoverflies) excluding honeybees. Summary of terms for generalized additive mixed models. ‡ Term was fitted using ti() function in GAMM. n=309.

| **Response variable**  **(Flower visitation**  **rate)** | **Parameter** | **Est. *df***  **(est. pp)** | **Effect^a^** | **Ref. df**  **(SE)** | **F-value  (t-value)** | **P** | **Deviance  explained  [%]** |
| --- | --- | --- | --- | --- | --- | --- | --- |
| **All** | (Intercept) | (2.03) | - | (0.13) | (15.69) | <0.001 | 26 |
| **pollinators** | Time of day^‡^ | 1.89 | quadratic | 1.89 | 12.42 | <0.001 |  |
| **without honeybees** | Flowering height^‡^ | 2.00 | quadratic | 2.00 | 10.05 | <0.001 |  |
|  | Plant species richness^‡^ | 2.00 | quadratic | 2.00 | 0.22 | 0.805 |  |
|  | Time of day * Flowering height^‡^ | 2.00 | quadratic | 2.00 | 0.86 | 0.426 |  |
|  | Time of day * Plant species richness | 1.00 | linear | 1.00 | 0.26 | 0.613 |  |
|  | Flowering height * Plant species richness | 1.00 | linear | 1.00 | 0.34 | 0.561 |  |
|  | Time of day * Flowering height *  Plant species richness^‡^ | 1.00 | linear | 1.00 | 0.13 | 0.721 |  |

## Figure S4: Effects of plant species richness, time of day and flowering height on flower visitation rate of the pollinator community without honeybees.

| (a)  **Without honeybees 4**   | (b)  **Without honeybees 60**   |
| --- | --- |
| **Figure S4**: Effects of plant species richness, time of day and flowering height on flower visitation rate of the pollinator community without honeybees. Shown are the results of minimal adequate generalized additive mixed models for all pollinator functional groups (bumblebees, solitary bees, hoverflies) without honeybees in mixtures with (a) four plant species and (b) 60 plant species: Flower visitation rate is not siginificantly influenced by plant species richness (4=low, 60=high plant species richness), but by time of day (0-1; range representing the observation time, between the onset of sunrise and sunset), and three different flowering heights (a-j: 1= 1-10 cm, 2= 11-25 cm, 3= ≥26 cm). | |

## Table S5: Plant species richness vs. flower cover as explanatory variable. Shown are the best models for all pollinator functional groups with either plant species richness (PSR) or flower cover (FC) as explanatory variable. Better model is indicated by Akaike information criterion with a correction for finite sample sizes (AICc) and numbers of degrees of freedom (df).

| **Modell** | **df** | **AICc** |
| --- | --- | --- |
| honeybees PSR  honeybees FC  bumblebees PSR  bumblebees FC  solitary bees PSR  solitary bees FC  hoverflies PSR  hoverflies FC | 23  23  13  23  23  23  16  23 | 1154.522  1201.246  1091.863  1142.916  1475.208  1443.503  1258.816  1275.898 |

## Table S6: All pollinator groups differed significantly in spatio-temporal (Flowering height and Time of day) resource use and in their response to plant species richness, as indicated by two-tailed Wald tests with bumblebees as a reference level; shown are p-values. The values 1, 2, 3 correspond to the knots; parameters in the table are weights of the B-spline basis functions, evaluated at knots 1, 2 or 3.

| \| **(Intercept)** \| **Honey**  **bees** \| **Hoverflies** \| **Solitary**  **bees** \| \| --- \| --- \| --- \| --- \| \| bs(Plant species richness)1 \| 0.408 \| 0.04 \| 0.036 \| \| bs(Plant species richness)2 \| 0 \| 0.739 \| 0.152 \| \| bs(Plant species richness)3 \| 0 \| 0.004 \| 0.001 \| \| bs(Flowering height)1 \| 0 \| 0 \| 0 \| \| bs(Flowering height)2 \| 0.819 \| 0.001 \| 0.015 \| \| bs(Flowering height)3 \| 0.819 \| 0.001 \| 0.015 \| \| bs(Time of day)1 \| 0.554 \| 0.015 \| 0.432 \| \| bs(Time of day)2 \| 0.706 \| 0.053 \| 0.119 \| \| bs(Time of day)3 \| 0.003 \| 0.538 \| 0.203 \| \| bs(Plant species richness)1:bs(Flowering height)1 \| 0.253 \| 0.033 \| 0.764 \| \| bs(Plant species richness)2:bs(Flowering height)1 \| 0 \| 0.003 \| 0.39 \| \| bs(Plant species richness)3:bs(Flowering height)1 \| 0.019 \| 0 \| 0.008 \| \| bs(Plant species richness)1:bs(Flowering height)2 \| 0 \| 0 \| 0 \| \| bs(Plant species richness)2:bs(Flowering height)2 \| 0 \| 0.003 \| 0.39 \| \| bs(Plant species richness)3:bs(Flowering height)2 \| 0.019 \| 0 \| 0.008 \| \| bs(Plant species richness)1:bs(Flowering height)3 \| 0 \| 0 \| 0 \| \| bs(Plant species richness)2:bs(Flowering height)3 \| 0 \| 0.506 \| 0.001 \| \| bs(Plant species richness)3:bs(Flowering height)3 \| 0 \| 0.012 \| 0 \| \| bs(Plant species richness)1:bs(Time of day)1 \| 0 \| 0 \| 0.002 \| \| bs(Plant species richness)2:bs(Time of day)1 \| 0 \| 0.115 \| 0.602 \| \| bs(Plant species richness)3:bs(Time of day)1 \| 0 \| 0 \| 0 \| \| bs(Plant species richness)1:bs(Time of day)2 \| 0 \| 0 \| 0 \| \| bs(Plant species richness)2:bs(Time of day)2 \| 0 \| 0.314 \| 0.416 \| \| bs(Plant species richness)3:bs(Time of day)2 \| 0.001 \| 0.553 \| 0.028 \| \| bs(Plant species richness)1:bs(Time of day)3 \| 0.86 \| 0.244 \| 0.044 \| \| bs(Plant species richness)2:bs(Time of day)3 \| 0 \| 0 \| 0 \| \| bs(Plant species richness)3:bs(Time of day)3 \| 0 \| 0 \| 0 \| \| bs(Flowering height)1:bs(Time of day)1 \| 0.029 \| 0 \| 0.004 \| \| bs(Flowering height)2:bs(Time of day)1 \| 0.996 \| 0.001 \| 0.016 \| \| bs(Flowering height)3:bs(Time of day)1 \| 0.996 \| 0.001 \| 0.016 \| \| bs(Flowering height)1:bs(Time of day)2 \| 0.955 \| 0.015 \| 0.72 \| \| bs(Flowering height)2:bs(Time of day)2 \| 0.006 \| 0.174 \| 0.062 \| \| bs(Flowering height)3:bs(Time of day)2 \| 0.006 \| 0.174 \| 0.062 \| \| bs(Flowering height)1:bs(Time of day)3 \| 0 \| 0.334 \| 0.001 \| \| bs(Flowering height)2:bs(Time of day)3 \| 0.487 \| 0.181 \| 0.873 \| \| bs(Flowering height)3:bs(Time of day)3 \| 0.487 \| 0.181 \| 0.873 \| |  |
| --- | --- | --- | --- | --- | --- | --- | --- | --- | --- | --- | --- | --- | --- | --- | --- | --- | --- | --- | --- | --- | --- | --- | --- | --- | --- | --- | --- | --- | --- | --- | --- | --- | --- | --- | --- | --- | --- | --- | --- | --- | --- | --- | --- | --- | --- | --- | --- | --- | --- | --- | --- | --- | --- | --- | --- | --- | --- | --- | --- | --- | --- | --- | --- | --- | --- | --- | --- | --- | --- | --- | --- | --- | --- | --- | --- | --- | --- | --- | --- | --- | --- | --- | --- | --- | --- | --- | --- | --- | --- | --- | --- | --- | --- | --- | --- | --- | --- | --- | --- | --- | --- | --- | --- | --- | --- | --- | --- | --- | --- | --- | --- | --- | --- | --- | --- | --- | --- | --- | --- | --- | --- | --- | --- | --- | --- | --- | --- | --- | --- | --- | --- | --- | --- | --- | --- | --- | --- | --- | --- | --- | --- | --- | --- | --- | --- | --- | --- | --- | --- |
| (continued) |  |
|  |  |

**Table S6.** (continued)

| **Honey Hoverflies Solitary**  **(Intercept) bees bees**   \| bs(Plant species richness)1:bs(Flowering height)1:bs(Time of day)1 \| 0.343 \| 0.084 \| 0.661 \| \| --- \| --- \| --- \| --- \| \| bs(Plant species richness)2:bs(Flowering height)1:bs(Time of day)1 \| 0 \| 0.004 \| 0.351 \| \| bs(Plant species richness)3:bs(Flowering height)1:bs(Time of day)1 \| 0 \| 0 \| 0 \| \| bs(Plant species richness)1:bs(Flowering height)2:bs(Time of day)1 \| 0.054 \| 0 \| 0 \| \| bs(Plant species richness)3:bs(Flowering height)3:bs(Time of day)1 \| 0 \| 0.006 \| 0.027 \| \| bs(Plant species richness)1:bs(Flowering height)1:bs(Time of day)2 \| 0.001 \| 0 \| 0.001 \| \| bs(Plant species richness)2:bs(Flowering height)1:bs(Time of day)2 \| 0 \| 0.063 \| 0 \| \| bs(Plant species richness)3:bs(Flowering height)1:bs(Time of day)2 \| 0 \| 0.287 \| 0.023 \| \| bs(Plant species richness)1:bs(Flowering height)2:bs(Time of day)2 \| 0 \| 0.414 \| 0 \| \| bs(Plant species richness)2:bs(Flowering height)2:bs(Time of day)2 \| 0 \| 0.063 \| 0 \| \| bs(Plant species richness)3:bs(Flowering height)2:bs(Time of day)2 \| 0 \| 0.287 \| 0.023 \| \| bs(Plant species richness)1:bs(Flowering height)3:bs(Time of day)2 \| 0 \| 0.414 \| 0 \| \| bs(Plant species richness)2:bs(Flowering height)3:bs(Time of day)2 \| 0 \| 0.707 \| 0 \| \| bs(Plant species richness)3:bs(Flowering height)3:bs(Time of day)2 \| 0 \| 0.005 \| 0 \| \| bs(Plant species richness)1:bs(Flowering height)1:bs(Time of day)3 \| 0.26 \| 0.25 \| 0.962 \| \| bs(Plant species richness)2:bs(Flowering height)1:bs(Time of day)3 \| 0 \| 0 \| 0 \| \| bs(Plant species richness)3:bs(Flowering height)1:bs(Time of day)3 \| 0 \| 0 \| 0 \| \| bs(Plant species richness)1:bs(Flowering height)2:bs(Time of day)3 \| 0 \| 0 \| 0 \| \| bs(Plant species richness)2:bs(Flowering height)2:bs(Time of day)3 \| 0 \| 0 \| 0 \| \| bs(Plant species richness)3:bs(Flowering height)2:bs(Time of day)3 \| 0 \| 0 \| 0 \| \| bs(Plant species richness)1:bs(Flowering height)3:bs(Time of day)3 \| 0 \| 0 \| 0 \| \| bs(Plant species richness)2:bs(Flowering height)3:bs(Time of day)3 \| 0 \| 0 \| 0.414 \| \| bs(Plant species richness)3:bs(Flowering height)3:bs(Time of day)3 \| 0.006 \| 0 \| 0.018 \| |
| --- | --- | --- | --- | --- | --- | --- | --- | --- | --- | --- | --- | --- | --- | --- | --- | --- | --- | --- | --- | --- | --- | --- | --- | --- | --- | --- | --- | --- | --- | --- | --- | --- | --- | --- | --- | --- | --- | --- | --- | --- | --- | --- | --- | --- | --- | --- | --- | --- | --- | --- | --- | --- | --- | --- | --- | --- | --- | --- | --- | --- | --- | --- | --- | --- | --- | --- | --- | --- | --- | --- | --- | --- | --- | --- | --- | --- | --- | --- | --- | --- | --- | --- | --- | --- | --- | --- | --- | --- | --- | --- | --- | --- |
|  |
|  |

## Figure S5: Effects of plant species richness on the flower visitation of all pollinators (honeybees, bumblebees, solitary bees, hoverflies), (a) based on data from Ebeling et al. (2008) and (b) based on our data

| 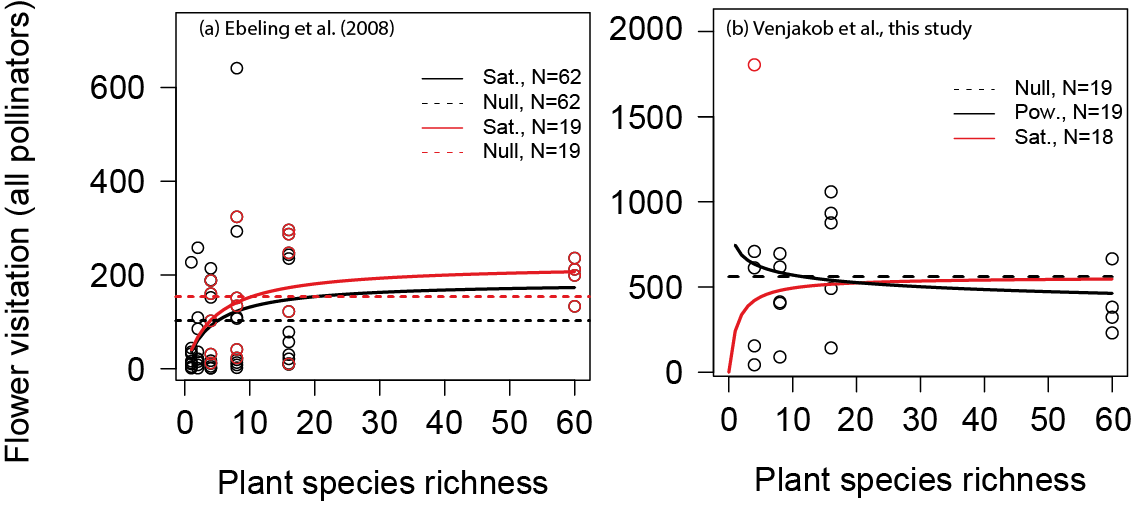 |
| --- |
| **Figure S5:** Effects of plant species richness on the flower visitation of all pollinators (honeybees, bumblebees, solitary bees, hoverflies), (a) based on data from Ebeling *et al.* (2008) and (b) based on our data. (a) solid lines are the predicted values of the saturated model (Sat.) and dotted lines show predictions from the null model (Null); red color indicates the number of observed plots (black = 62 plots; red = 19 plots, exactly the same plots as used in our experiment). (b) black solid line shows predicted values of the power model (Pow., 19 plots) with an outlier (red circle), red solid line shows prediction of the saturated model (Sat., 18 plots) without the outlier, dotted line shows predictions from the null model (Null, 19 plots).  Figure (a) shows that effects of plant species richness on flower visitation were not fundamentally altered by our sampling design with N=19 plots. Figure (b) shows that plant species richness had a positive effect on the flower visitation of all pollinators resulting in a saturation curve as found in Ebeling *et al.* (2008). |

# References

1.

Bivand, R. & Lewin-Koh, N. (2015). maptools: Tools for Reading and Handling Spatial Objects.

2.

Bolker, B.M. (2013). [R-sig-ME] How to calculate proportion of deviance explained from GAMM? Available at: https://stat.ethz.ch/pipermail/r-sig-mixed-models/2013q4/021389.html. Last accessed 7 February 2014.

3.

Ebeling, A., Klein, A.-M., Schumacher, J., Weisser, W.W. & Tscharntke, T. (2008). How does plant richness affect pollinator richness and temporal stability of flower visits? *Oikos*, 117, 1808–1815.

4.

Robinson, R. (2013). [R-sig-ME] How to calculate proportion of deviance explained from GAMM? Available at: https://stat.ethz.ch/pipermail/r-sig-mixed-models/attachments/20131203/3fe5ef24/attachment.pl. Last accessed 7 February 2014.
